# Novel Immunomodulators from Hard Ticks Selectively Reprogramme Human Dendritic Cell Responses

**DOI:** 10.1371/journal.ppat.1003450

**Published:** 2013-06-27

**Authors:** Stephen G. Preston, Juraj Majtán, Chrisoula Kouremenou, Oliwia Rysnik, Lena F. Burger, Alejandro Cabezas Cruz, Maylin Chiong Guzman, Miles A. Nunn, Guido C. Paesen, Patricia A. Nuttall, Jonathan M. Austyn

**Affiliations:** 1 Nuffield Department of Surgical Sciences, University of Oxford, John Radcliffe Hospital, Oxford, United Kingdom; 2 Department of Zoology, University of Oxford, Oxford, United Kingdom; 3 Institute of Zoology, Slovak Academy of Sciences, Bratislava, Slovakia; 4 Nuffield Department of Clinical Medicine, University of Oxford, John Radcliffe Hospital, Oxford, United Kingdom; 5 University of South Bohemia, Faculty of Science and Biology Centre of the ASCR, Institute of Parisitology, České Budějovice, Czech Republic; 6 Center for Genetic Engineering and Biotechnology, Animal Biotechnology Division, Havana, Cuba; 7 NERC Centre for Ecology & Hydrology, Crowmarsh Gifford, Wallingford, Oxfordshire, United Kingdom; 8 Division of Structural Biology, Henry Wellcome Building for Genomic Medicine, Oxford, United Kingdom; Medical College of Wisconsin, United States of America

## Abstract

Hard ticks subvert the immune responses of their vertebrate hosts in order to feed for much longer periods than other blood-feeding ectoparasites; this may be one reason why they transmit perhaps the greatest diversity of pathogens of any arthropod vector. Tick-induced immunomodulation is mediated by salivary components, some of which neutralise elements of innate immunity or inhibit the development of adaptive immunity. As dendritic cells (DC) trigger and help to regulate adaptive immunity, they are an ideal target for immunomodulation. However, previously described immunoactive components of tick saliva are either highly promiscuous in their cellular and molecular targets or have limited effects on DC. Here we address the question of whether the largest and globally most important group of ticks (the ixodid metastriates) produce salivary molecules that specifically modulate DC activity. We used chromatography to isolate a salivary gland protein (Japanin) from *Rhipicephalus appendiculatus* ticks. Japanin was cloned, and recombinant protein was produced in a baculoviral expression system. We found that Japanin specifically reprogrammes DC responses to a wide variety of stimuli in vitro, radically altering their expression of co-stimulatory and co-inhibitory transmembrane molecules (measured by flow cytometry) and their secretion of pro-inflammatory, anti-inflammatory and T cell polarising cytokines (assessed by Luminex multiplex assays); it also inhibits the differentiation of DC from monocytes. Sequence alignments and enzymatic deglycosylation revealed Japanin to be a 17.7 kDa, N-glycosylated lipocalin. Using molecular cloning and database searches, we have identified a group of homologous proteins in *R. appendiculatus* and related species, three of which we have expressed and shown to possess DC-modulatory activity. All data were obtained using DC generated from at least four human blood donors, with rigorous statistical analysis. Our results suggest a previously unknown mechanism for parasite-induced subversion of adaptive immunity, one which may also facilitate pathogen transmission.

## Author Summary

Dendritic cells (DC) are specialised cells of the vertebrate immune system. DC can sense different types of infectious agents and parasites, and both trigger and help regulate the specific types of immunity needed to eliminate them. We have discovered that the largest and globally most important group of hard ticks produce a unique family of proteins in their saliva that selectively targets DC, radically altering functions that would otherwise induce robust immune responses; these proteins also prevent DC developing from precursor cells. The production of these salivary molecules may help to explain two highly unusual features of these hard ticks compared with other blood-feeding parasites: their ability to feed continuously on their vertebrate hosts for considerable lengths of time (7 days or more) without eliciting potentially damaging immune responses, and their capacity to transmit possibly the greatest variety of pathogens of any type of invertebrate.

## Introduction

Hard ticks (Ixodidae) adopt a feeding strategy in which they cut into the skin of their hosts to insert their mouthparts, then remain attached for extended periods (in the case of adult females, a week or more) taking a single, large blood meal. This makes them unique amongst blood-feeding arthropods (such as mosquitoes and sand flies) which otherwise feed little and often, with each meal lasting just minutes [1], [2]. In order to feed successfully, hard ticks must somehow overcome not only haemostasis and the rapidly-responding components of innate immunity, but also the slower-developing adaptive immune response of their vertebrate hosts. Their apparent ability to overcome these challenges and to subvert host immunity may help explain why they transmit possibly the greatest diversity of pathogens of any arthropod vector. For example, *Rhipicephalus appendiculatus* (the brown ear tick) transmits the protozoan *Theileria parva*, the causative agent of the devastating cattle disease East Coast fever, and Nairobi sheep disease virus which causes severe disease in sheep and goats; *R. sanguineus* (the brown dog tick) transmits the bacterium *Rickettsia conori*, causing Mediterranean spotted fever in humans; *R. (Boophilus) microplus* (the cattle tick) is globally the most important tick parasite of livestock, transmitting babesiosis and anaplasmosis infections; and *Dermacentor andersonii* (the Rocky Mountain wood tick) transmits the bacterium causing Rocky Mountain spotted fever, the most lethal form of rickettsial illness in humans.

Innate immunity is triggered primarily by evolutionary-conserved features of pathogen-derived molecules, or by the molecular signatures of tissue damage or stress [3]. These are typically detected by pattern recognition receptors (PRRs) on tissue-resident cells, such as mast cells and macrophages, as well as soluble PRRs in the tissue fluids. The former include Toll-like receptors (TLRs), while the latter include components that activate the complement cascade. A major outcome of both TLR and complement activation is the initiation of an inflammatory response. Locally, this results both in increased vascular permeability, with the movement of soluble effector molecules to the site of insult and, importantly, in further recruitment of innate effector cells such as neutrophils and monocytes into the tissue. Hard ticks appear to protect themselves from the effector molecules of innate immunity, in part by producing a diversity of proteins that bind to and neutralise soluble mediators, such as mast cell-derived histamine and complement components [4]–[7]. They also possess mechanisms to limit the development of the inflammatory response in the shape of “evasins” which bind to and neutralise chemokines, the primary mediators of leucocyte recruitment [8], as well as proteins that appear to inhibit neutrophil function [9]. In contrast, adaptive immunity is mediated by two types of lymphocyte, T cells and B cells, with the former being broadly divided into CD4^+^ and CD8^+^ T cells. CD4^+^ T cells orchestrate the immune response by recruiting, activating, and regulating other effector cells (including those of innate immunity), whereas CD8^+^ T cells develop into cytotoxic T cells which eliminate cells with intracellular infections, and B cells differentiate into antibody-secreting plasma cells. To counter these responses, components of tick saliva and salivary gland extracts (SGEs) from hard ticks can inhibit adaptive immunity by inhibiting lymphocyte function or by binding and neutralising antibodies [10]–[13].

Dendritic cells (DC) bridge innate and adaptive immunity. DC are the key initiators and modulators of T cell responses, and so play pivotal roles in the initiation and regulation of adaptive immunity as a whole [14]–[16]. They are resident within most peripheral tissues, including the skin, and act as immune “sentinels” which sample antigens from their surroundings for subsequent recognition by T cells (antigen presentation), whilst also detecting “danger”, in the shape of pathogens or tissue damage, through their expression of PRRs [14], [15]. In response to such stimuli, DC undergo a programme of phenotypic and functional changes termed maturation, during which they also migrate from the periphery into secondary lymphoid tissues. Here, they activate naïve antigen-specific T cells [16]. DC are uniquely effective in doing so through their capacities both to degrade protein antigens to peptides for loading onto MHC Class I or II molecules (for recognition by CD8^+^ and CD4^+^ T cells, respectively) and to express the specialised “co-stimulatory” molecules which are required for T cell activation. Their influence on the T cell response is not however simply limited to its initiation. Following activation, CD4^+^ T cells may differentiate into different subclasses of effector cells (notably Th17, Th1 and Th2 cells, each of which drives the appropriate immune response for the elimination of a different class of threat), or into regulatory T cells (Treg), which can contribute to a state of antigen-specific immunological non-responsiveness or tolerance. DC direct this differentiation, through factors which include their profile of co-stimulatory molecule expression and their secretion of T cell-polarising cytokines [15], [17].

It is clear that manipulation of DC provides a mechanism by which a parasite or pathogen could profoundly affect the adaptive immune response, either by inhibiting the response completely (for example, by preventing DC activation of T cells entirely, or by driving Treg differentiation) or misdirecting it, thus resulting in the generation of a type of adaptive response that is ineffectual at repelling the invader. Given this, it is no surprise that many parasites (including viruses, bacteria, protozoa, and metazoa) have evolved strategies to modulate DC function [18]–[22]. The same also seems true of hard ticks, which are classified into two main groups, the metastriates and prostriates, with the former representing the majority of known species [23]. Both groups elaborate broad-spectrum immune modulators which also have effects on DC: prostriate ticks produce prostaglandin E2 (PGE2), while metastriate ticks produce PGE2, adenosine, and Sialostatin L [24]–[26]. However, only in prostriates has a modulatory protein been identified which acts on DC with any degree of specificity. This protein, Salp15, inhibits pro-inflammatory cytokine secretion by DC whilst additionally modulating CD4^+^ T cell function [27], [28]. To our knowledge, no such molecule has been reported in any metastriate species. We therefore hypothesised that metastriate ticks may have evolved distinctive DC-modulatory proteins. Here we report the identification and characterisation of a unique class of proteins specific to metastriate ticks. These molecules selectively and profoundly modulate the maturation of DC in response to diverse stimuli, and prevent their development from precursors.

## Results

### *Rhipicephalus appendiculatus* salivary glands produce a DC-modulatory protein, Japanin

To search for DC modulators produced by metastriate ticks we designed a simple screen, based on the capacity of tick salivary gland extracts (SGE) to modulate DC maturation in response to bacterial lipopolysaccharide (LPS); LPS was employed as it is by far the best studied DC maturation stimulus. We initially focused our efforts on studying SGE from *Rhipicephalus appendiculatus*, the vector of *Theileria parva*. Cattle are the preferred hosts of *R. appendiculatus* at all life stages, but collections have also been taken from other large mammals, including humans. Rodents, however, do not appear to be good hosts for any life stage [29]. We employed human DC throughout this study.

To screen for the presence of DC modulators, we first treated monocyte-derived DC with SGE from adult *R. appendiculatus*, then added LPS as a model stimulus. LPS acts as an agonist for TLR4 on DC and triggers their maturation, the extent of which can be assessed by determining surface levels of the co-stimulatory molecule CD86, which is reliably increased (“upregulated”) during normal DC maturation. DC-modulatory activity in SGE was measured as a reduction in CD86 upregulation in response to LPS. Such an activity was indeed observed following incubation with SGE from 3 day-fed females, but not after incubation with SGE from unfed or 6-day fed females, nor with SGE from any males (figure S1). SGE from 3 day-fed females (SGE-3F) was therefore selected for further study.

Treatment of SGE-3F with Proteinase K abrogated its DC-modulatory activity, while mock-treatment had no effect, showing that a proteinaceous SGE component was sufficient for DC modulation in this assay (figure S2). We cannot entirely exclude contributions by PGE2 or adenosine, both previously described as non-protein immunomodulatory components of tick saliva (see above), but the failure of mock-treatment (using the same buffers and temperatures) to substantially reduce activity shows that any such contribution must relatively small compared with the effects of the active protein component(s). Furthermore, the complete abrogation of activity in Proteinase K samples suggests that any non-protein active components are labile under the treatment conditions (i.e. due to thermal instability). Prostaglandin E2 is unstable in aqueous solution, particularly at high temperatures [30] and may have been destroyed by treatment. Adenosine, however, would be expected to survive heat treatment, so our results suggest that it is not present in significant quantities in SGE-3F. Previous studies have described adenosine in saliva from 5–7 day fed *R. sanguineus* [26], but we are not aware of any reports of its presence in saliva, or in SGE, after 3 days of feeding. Its presumed absence from our samples may, therefore, be attributable to the length of feeding, or to the use of SGE rather than saliva.

Multiple rounds of chromatography were then used to isolate the active protein. SGE-3F was first passed through a Q column at pH7.0, removing many SGE components but leaving the DC-modulatory activity intact. The Q column flow-through was then fractionated using size exclusion chromatography, and a fraction with DC-modulatory activity was further fractionated using HPLC. Activity was detected in a group of consecutive fractions, centred around a single peak on the HPLC trace, from which Edman sequencing generated a 16 residue N-terminal sequence: TPSMPAINTQTLYLAR.

We used the above N-terminal sequence to design degenerate primers for polymerase chain reaction (PCR) cloning of a 460 bp sequence from *R. appendiculatus* salivary gland cDNA (data not shown). This sequence (representing the 3′ region of the candidate mRNA) was, in turn, employed to design primers for amplification of the 5′ region using 5′RACE. Finally, we performed PCR cloning of the complete coding sequence of the putative DC-modulatory protein, which we named “Japanin” (Genbank accession KC412662). Its 531 bp coding sequence encodes a 176 amino acid peptide. Analysis with SignalP 4.0 [31] suggests that it is a secretory protein, lacking a transmembrane domain, and comprising a 24 residue cleavable signal peptide followed by a 152 residue mature peptide of predicted 17.7 kDa molecular weight, the N-terminal sequence of which is consistent with that obtained by Edman degradation. Inspection of the primary amino acid sequence of Japanin indicated that it is a member of the lipocalin family (see below).

To facilitate detection and purification of recombinant Japanin, we constructed a polyhistidine-tagged version using PCR. This “Japanin-his” DNA sequence (comprising a Kozak consensus sequence for initiation of translation [32], the full-length Japanin coding sequence, and a tag sequence encoding a diglycine linker and six histidine residues) was subcloned into bacterial and mammalian expression vectors (pET52b and pCDNA3.1), and into a transfer vector (pBacPAK8) for the generation of recombinant baculovirus (see materials and methods). The polyhistidine tag enabled the detection of recombinant Japanin in Western blots with an anti-polyhistidine antibody. We used this to show that Sf9 cells infected with Japanin-His-baculovirus secreted recombinant Japanin, as did pcDNA3.1-Japanin-His transfected HEK293T and CHO cells. We were not, however, able to recover intact recombinant Japanin from bacterial expression cultures (data not shown).

Since it seemed more appropriate to use an arthropod, rather than a mammalian, system for expression of a tick protein, Sf9-derived Japanin was used in subsequent experiments. It was isolated from the supernatant of Sf9 expression cultures by binding to Talon resin, and further purified with a gel filtration polishing step (see Materials and Methods).

**Figure 1:**
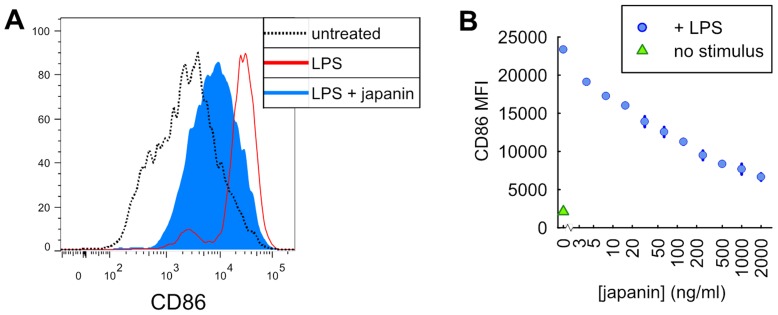
Pretreatment of dendritic cells with Japanin inhibits their upregulation of CD86 in response to LPS. Dendritic cells were incubated with japanin for 24 hours prior to the addition of LPS (100 ng/ml) for a further 18–20 hours. CD86 expression was then analysed by flow cytometry. (A) The results from a representative experiment using 500 ng/ml japanin. (B) Titration of Japanin concentration, showing a dose-dependent inhibition of CD86 upregulation. The range and mean of duplicate measurements from one representative experiment are shown. This experiment was performed four times, with dose-dependency demonstrated each time, but with EC_50_ varying between donors.

**Figure 2:**
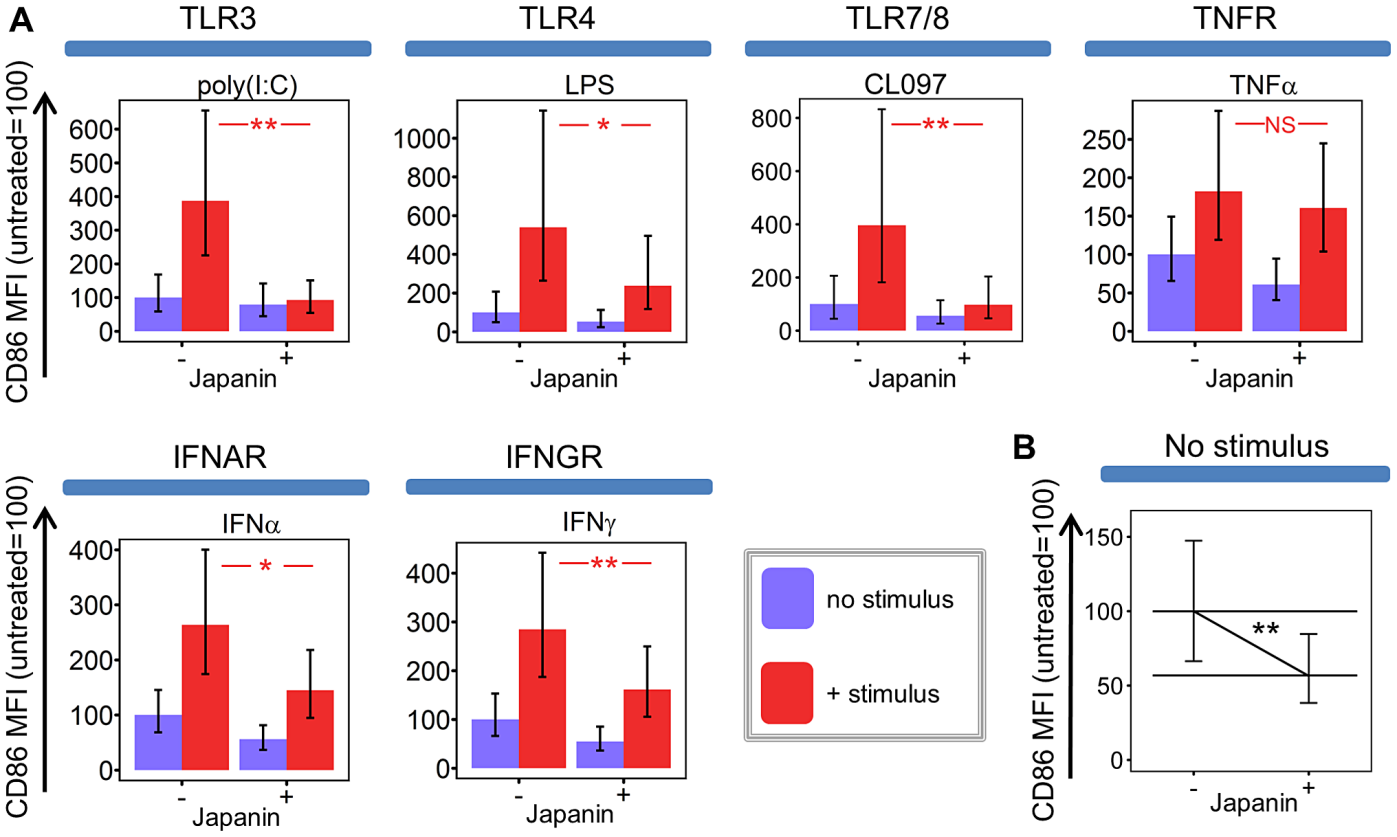
Japanin inhibits CD86 upregulation in response to multiple DC maturation stimuli. (A) Dendritic cells were cultured for 18–20 hours in the presence or absence of Japanin (500 ng/ml) and stimuli: (25 µg/ml Poly(I:C), via TLR3; 100 ng/ml LPS, TLR4; 4 µg/ml CL097, TLR7/8; 20 ng/ml IFNα2, IFNAR; 10–12.5 ng/ml TNFα, TNFR; 20 ng/ml IFNγ, IFNGR). CD86 expression was then assessed by flow cytometry. Modelled means ±95% confidence intervals using data from at least four experiments are shown, except for CL097 for which three experiments were performed, using cells from a total of five donors. (B) Data from all these experiments was used to assess the effect of Japanin on CD86 expression in the absence of stimuli. ** p<0.01, * p<0.05, NS p>0.05.

In order to confirm that we had indeed identified a protein with DC-modulatory properties, we employed the same assay previously used to screen SGE, assessing the ability of Japanin to inhibit DC upregulation of CD86 in response to LPS. To establish whether any effect was dose-dependent, we tested the effect of Japanin at a range of concentrations. As can be seen from the results of a representative experiment in figure 1, Japanin had a potent and dose-dependent effect on DC maturation, although responses to any given dose differed between donors (not shown); figure 2a (see TLR4) shows analysis of the data from 20 independent experiments in which CD86 upregulation was reduced by an average of around 50% by 500 ng/ml Japanin.

That Japanin was apparently produced only by three day-fed female ticks amongst the cohorts examined is not entirely surprising, as temporal regulation and gender differences in tick saliva proteins are well-described phenomena [33]. Hard tick feeding occurs in two stages: slow and rapid [34]. In adult females, slow feeding lasts 6 to 7 days or more with a 10-fold weight gain; rapid feeding lasts only a further 12–24 hours during which body weight increases a further 10-fold [35]. These distinct feeding stages may explain why Japanin is present in 3 day-fed female SGE (from the slow-feeding stage) but apparently not in 6 day-fed SGE (from the rapid-feeding stage). Likewise, the initiation of feeding is required for de novo production of many factors in tick saliva, so the absence of DC modulators in SGE from unfed ticks was unsurprising. The absence of DC modulatory activity in male tick SGE at all time-points may be related to the fact that they take a smaller blood meal than females [1] and so may have less need to modulate DC function and, potentially, the host's adaptive immune response. In fact, there are numerous reports of differences between conspecific male and female ticks in SGE activities, for example in immunoglobulin-binding proteins [36], histamine binding proteins [4], and chemokine binding proteins [37]. One possible reason is that females focus on imbibing an enormous blood meal to produce thousands of eggs, increasing in size a hundred-fold, whereas male *R. appendiculatus* demonstrate ‘mate guarding’ by secreting male-specific immunomodulatory proteins that help their mate to feed [36].

### Japanin specifically binds to dendritic cells

**Figure 3:**
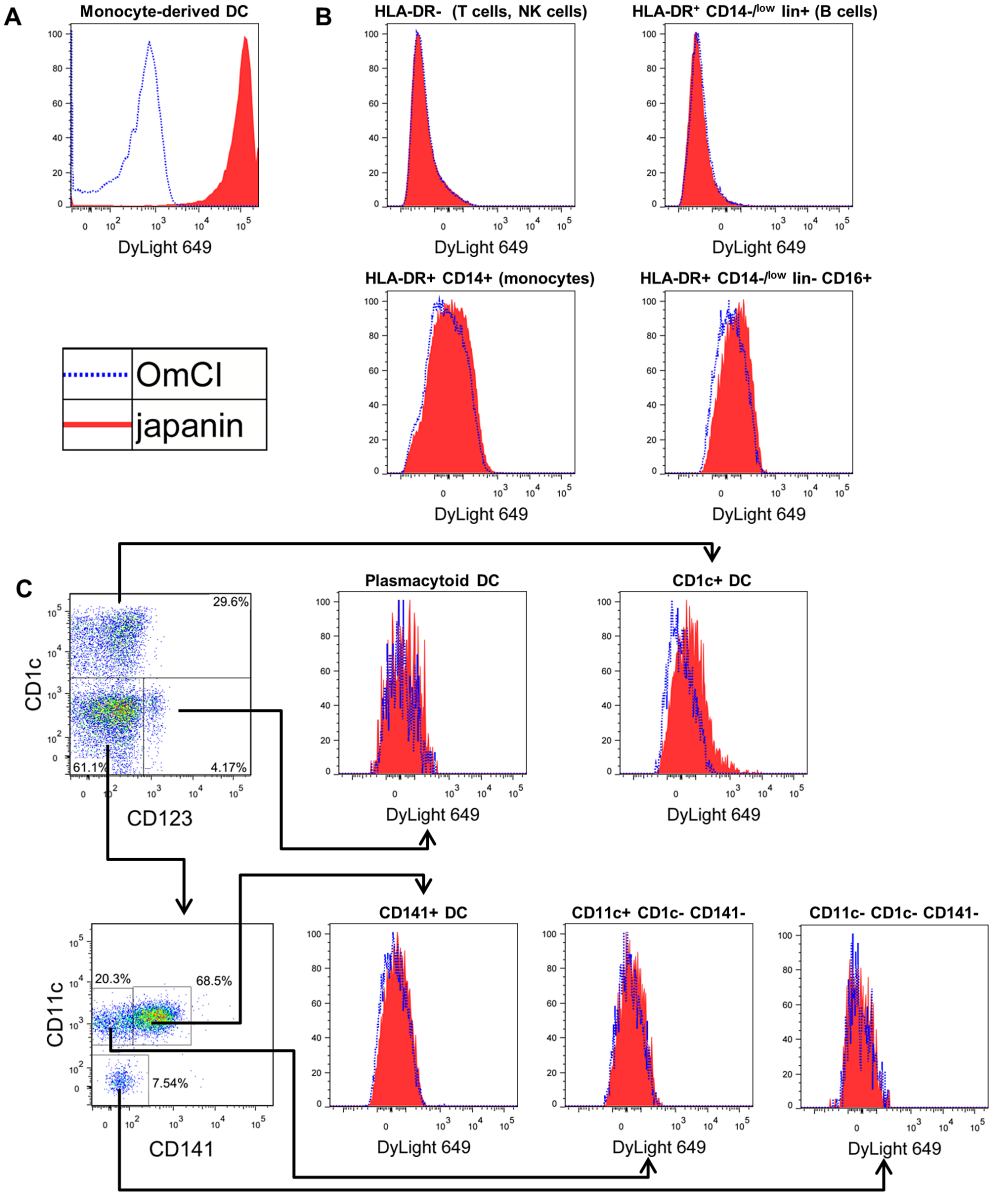
Japanin binds specifically to DC. (A) Monocyte-derived dendritic cells, or (B and C) human blood PBMC were incubated with 100 ng/ml Japanin-DyLight 649 (filled histograms) or 340 ng/ml OmCI-DyLight 649 (open histograms), incubated on ice for 1 hour, and washed. Binding was assessed by flow cytometry. In B, major PBMC subsets are defined by surface molecule expression. In C, blood DC are defined as CD14^−^HLA-DR^+^lineage^−^CD16^−^ then further subdivided according to CD1c, CD11c, CD123 and CD141 expression. For the complete gating strategy, see figure S4. Results shown are representative of those from 6 (A) and 4 (B and C) experiments with cells from different donors.

Having shown that Japanin has DC-modulatory properties, we next assessed whether or not it binds, and potentially acts, specifically on DC. We labelled recombinant Japanin with a fluorochrome, and measured its binding to monocyte-derived DC and to peripheral blood mononuclear cell subsets (PBMC) by flow cytometry. Fluorochrome-labelled OmCI (a tick-derived lipocalin with no known effect on DC [38]) was used as a control for non-specific binding. Japanin bound strongly to monocyte-derived DC (figure 3a) and at a low level to CD1c^+^ DC (figure 3c) but there was no appreciable binding to any major populations in PBMC (figure 3b), including monocytes (defined as lin^−^CD14^+^ cells), B cells (lin^+^HLA-DR^+^CD14^−^), T cells or NK cells (lin^+^HLA-DR^−^ cells). Nor was there appreciable binding to other blood DC subsets (figure 3c), or to activated T cells that had been stimulated for 4 days with anti-CD3/CD28 beads (figure S3). Gating strategies are shown in figure S4. These data clearly show Japanin to be a highly-specific DC modulator, the first described from a tick. Notably, it does not bind to T cells, even after their activation, suggesting that it could potentially influence adaptive immunity solely by acting on DC. The results distinguish Japanin from the prostriate tick-derived Salp15, which binds to both DC through DC-SIGN, and T cells through CD4, directly modulating the activity of both cell types [28].

### Japanin reprogrammes dendritic cell maturation

DC maturation is a complex process which can endow the cells with both stimulatory and inhibitory functions. Classically, it involves upregulation of co-stimulatory molecules, such as CD86, which help to initiate adaptive responses, along with the secretion of pro-inflammatory cytokines and T cell-polarising cytokines. DC may also upregulate co-inhibitory molecules, such as CD274 (B7H1; PD-L1), which help to suppress adaptive responses, and secrete anti-inflammatory cytokines or express alternate T cell-polarising molecules. The balance between these various responses is determined in part by the nature and duration of the maturation stimulus, including any intrinsic host-derived DC-modulating factors, and these in turn help to determine the strength and nature of the subsequent T cell response and the overall type of immunity that results.

Being a prokaryotic product, LPS is not produced by ticks. We reasoned that Japanin was therefore not likely to have evolved as a specific regulator of LPS-induced maturation, and hypothesised that it may also modulate DC maturation in response to other stimuli. We found that Japanin was capable of inhibiting CD86 upregulation in response to a wide range of stimuli including the TLR3 agonist poly(I:C) and the TLR7/8 agonist CL097, as well as the cytokines IFN-α2 and IFN-γ which signal through entirely distinct intracellular pathways (figure 2a). Preliminary studies further suggested that Japanin inhibits CD86 upregulation in response to the TNF-family member CD154 (CD40L ligand) which is crucial for cross-talk between DC and activated T cells (data not shown). In fact, the only stimulus tested for which Japanin did not induce a clear inhibition of CD86 upregulation was TNF-α (figure 2a). Furthermore, the modulatory activity of Japanin is not based on interruption of receptor-proximal signalling components, as these are not shared between TLR and IFN-receptor signalling pathways [39].

Given that the role of DC in adaptive immunity is not limited to activation, but extends to educating and directing the T cell response, we speculated that the tick might benefit more from subverting DC maturation than from simply inhibiting it; for example this might result in the development of a type of immunity that is harmless to the tick, or even in the induction of tolerance. To investigate whether Japanin had effects more sophisticated than a simple inhibition of CD86 expression, we extended our studies to the expression of other membrane molecules associated with DC maturation (using flow cytometry), and to the secretion of a wide variety of cytokines (using multiplex analysis of culture supernatants). In these experiments, we added Japanin at the same time as LPS, rather than as a pre-treatment, as trial experiments showed that this reduced intra-experimental variance in cytokine concentration readings (data not shown).

**Figure 4:**
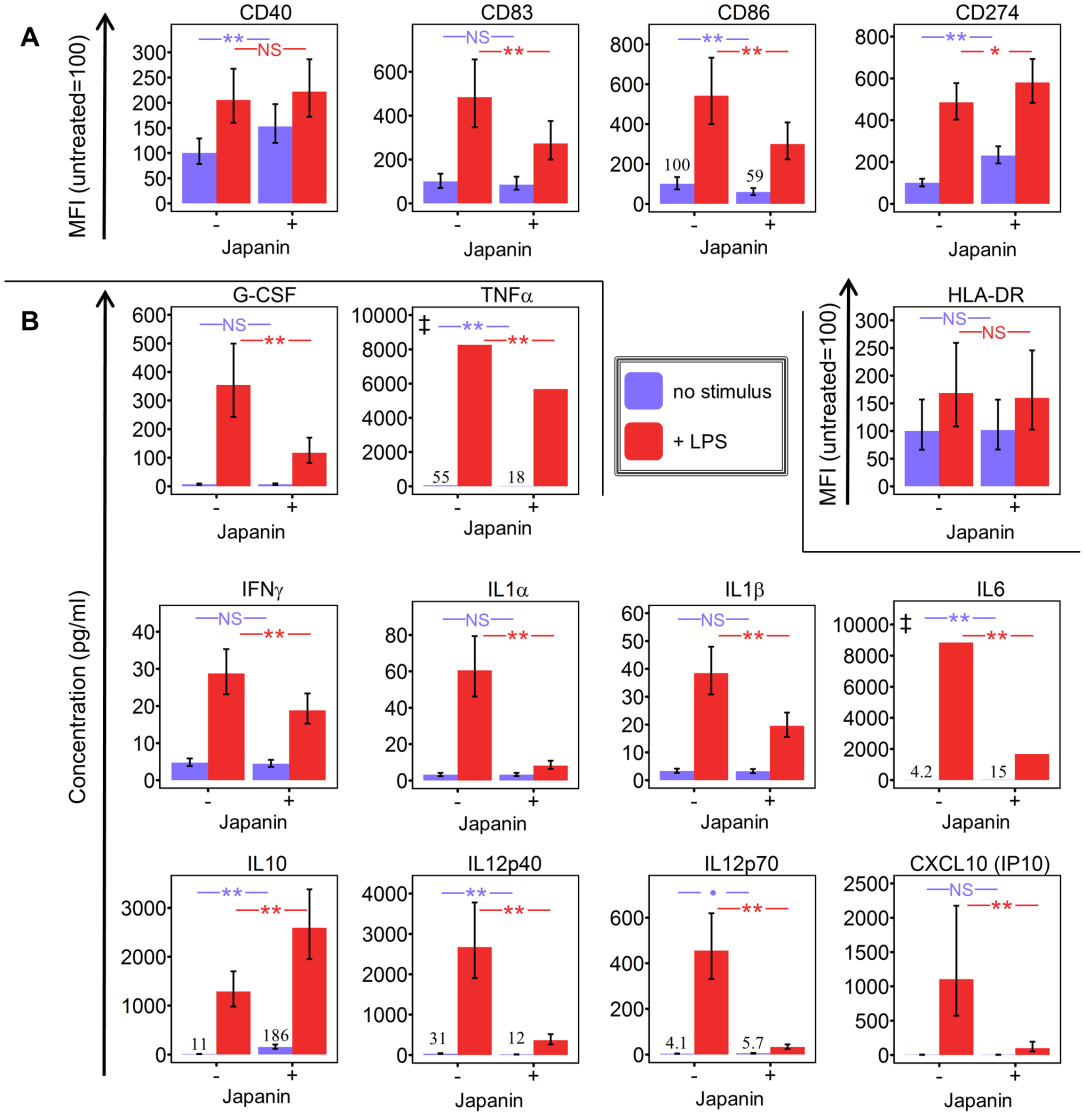
Japanin modulates dendritic cell maturation, rather than simply inhibiting it. Dendritic cells were cultured in the presence or absence of Japanin (500 ng/ml) and LPS (100 ng/ml) for 18–20 hours. (A) CD40, CD83, CD86, CD274 and HLA-DR expression were then assessed by flow cytometry, and (B)the concentration of pro-inflammatory cytokines in the culture supernatant was measured by Luminex1. Modelled means ±95% confidence intervals using data from at least four experiments are shown, except where marked ‡ where above-scale readings in the LPS-only made it impossible to calculate meaningful confidence intervals; the graphs show the lowest possible mean value (taking an above-scale value to be equal to the maximum possible on-scale value). ** p<0.01, * p<0.05, 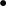 p<0.1, NS p>0.05.

We found that the effects of Japanin extend to a marked reduction in the upregulation of not just CD86 but also the maturation marker CD83 (figure 4a) and to a dramatic reduction in the secretion of a range of cytokines. The latter included pro-inflammatory [IL-1-β, IL-6, TNF-α] and/or Th17-polarising [IL-1-β, IL-6] and Th1-polarising [IL-12p70, IFN-γ] molecules (figure 4b). However, this was not due to a complete inhibition of DC maturation, as Japanin *enhanced* expression both of the co-inhibitory molecule CD274 (figure 4a) and of the anti-inflammatory cytokine IL-10 (figure 4b). Moreover, Japanin had no significant effect on expression of MHC Class II molecules (HLA-DR) or the co-stimulatory molecule CD40 (figure 4a), which are respectively required for antigen presentation to, and cross-talk with, T cells. Nor did it have a significant effect on LPS-induced secretion of IL-7, IL-8 or CCL11 (figure S5). Interestingly, Japanin was also active in the absence of LPS, reducing CD86 expression, increasing CD274 (as well as CD40) expression, and enhancing IL-10 secretion (figures 2b, 4a, 4b) during the ‘spontaneous’ DC maturation that occurred slowly while in culture. Collectively, the above results show that Japanin acts through a sophisticated reprogramming of the DC maturation process, rather than by simply blocking it. Such a complex spectrum of effects has never previously been reported for a DC modulator (see discussion) suggesting that Japanin affects a distinct and novel transcriptional programme in DC.

### Japanin arrests dendritic cell development from monocytes

**Figure 5:**
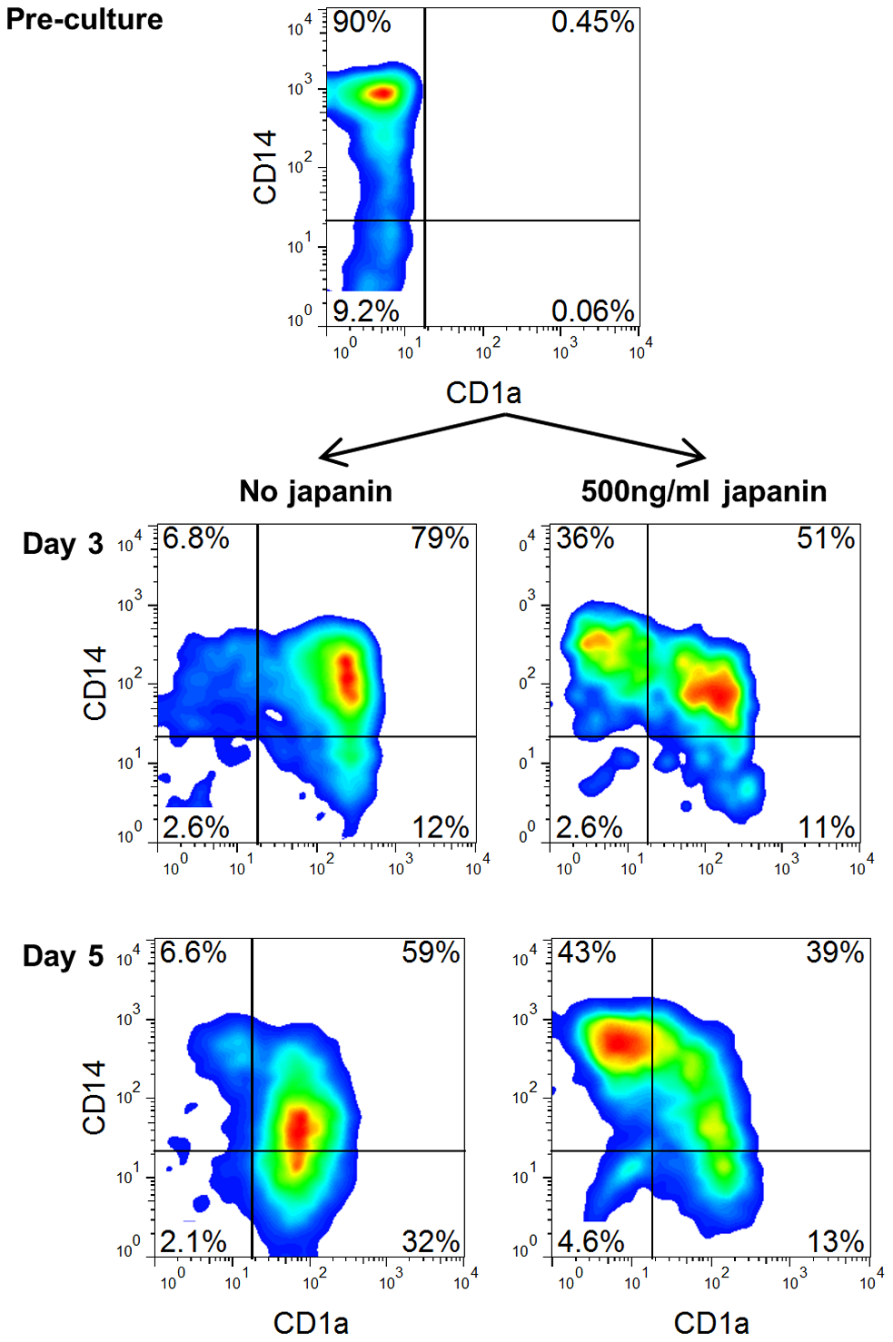
Japanin blocks differentiation of DC from monocytes. Monocytes were cultured with GM-CSF (1000 U/ml) and IL4 (500 U/ml) with or without Japanin (500 ng/ml). Before the culture, and again after 3 and 5 days of culture, CD1a and CD14 expression were assessed by flow cytometry, in order to monitor differentiation into CD1a^+^CD14^low^ dendritic cells. Data shown is from one experiment, representative of three independent experiments using cells from different donors.

Following maturation in response to injury or other stimuli, skin-resident DC typically migrate out of the skin into the lymphatics and move to the draining lymph nodes. This can happen extremely rapidly, and involve the large majority of skin DC, potentially resulting in a situation where an exodus of pre-existing DC could leave the skin effectively DC-free and “unguarded” [40], [41]. In order to avert this, monocytes are recruited from the blood into sites of inflammation where they are capable of differentiating into DC, thus replenishing the DC population and restoring immune monitoring [42], [43]. It would seem to be advantageous to the tick to be able to prevent such replenishment, and so we looked at the ability of Japanin to affect the differentiation of DC from monocytes in vitro. To do this, we employed the same system which we used previously for the generation of DC from monocytes in the presence of GM-CSF and IL-4, but this time added Japanin to the differentiation cultures from the start. In the absence of Japanin, these conditions typically promoted the development of CD14^high^ CD1a^−^ monocytes into CD14^low^ CD1a^+^ DC. However we found that when Japanin was added ∼50% of cells retained the CD14^high^CD1a^−^ monocyte-like phenotype, even after 5 days in culture (figure 5).

The above results appear to conflict with the finding that Japanin does not bind monocytes. However, we found that binding of Japanin to monocytes increases during culture with GM-CSF and IL-4 (figure S6), suggesting that it could act during the early stages of differentiation of monocytes into DC. Japanin therefore seems to impose a powerful block on this differentiation process. Given the influx of monocytes into the bite site in response to local tissue damage [44], the effect of Japanin on differentiating monocytes may be as important to ticks as its effects on DC, preventing the re-establishment of immune surveillance following initial DC exodus. Further study of the Japanin-treated cells will be required to elucidate whether they truly resemble “arrested” monocytes, or whether they have differentiated along an alternative pathway, but what is clear is that DC differentiation is blocked or greatly altered.

### Japanin is a lipocalin

**Figure 6:**
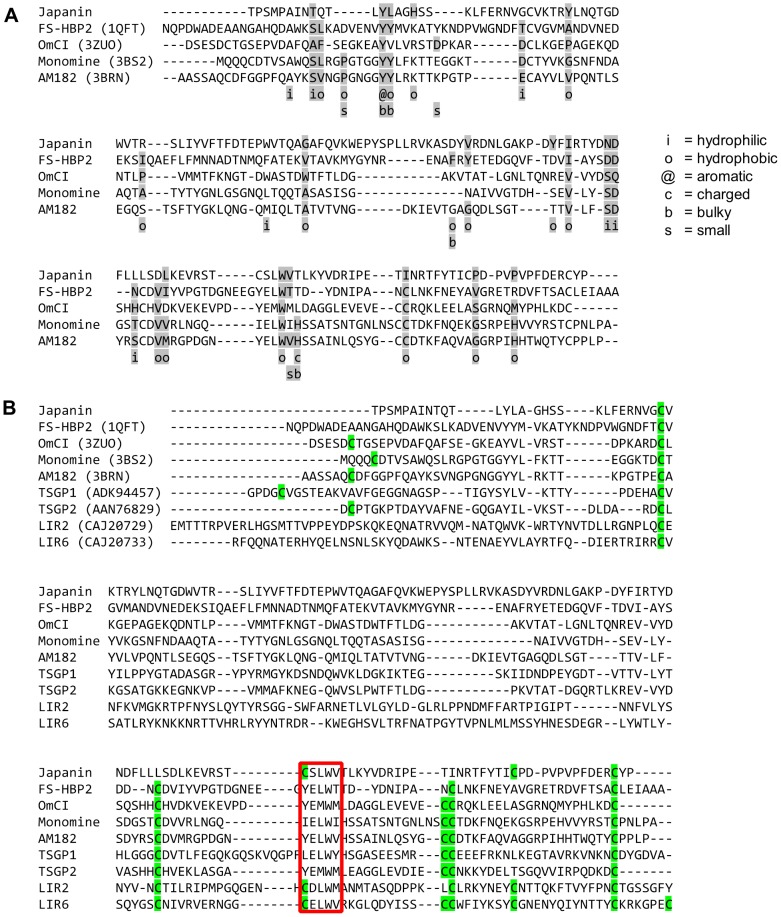
Japanin is a lipocalin. The mature Japanin sequence was aligned with (A) sequences of mature tick proteins with a resolved lipocalin structure, or (B) with these and additional distantly-related sequences also accepted to be tick lipocalins. In A, key residues identified by Adam and colleagues [47] are shaded, and their nature noted below. Residue characteristics are: hydrophobic = ACFGHILMPVWY; hydrophilic = DEHKNQRSTY; charged = DEHKR; aromatic = FHWY; bulky = EFHIKLMQRWY; small = not bulky. In B, cysteine residues are highlighted green, and the conserved tick lipocalin motif is boxed red.

Lipocalins are a family of small (∼20 kDa) proteins characterised by an eight-stranded antiparallel β-barrel fold with a repeated +1 topology, typically preceded by a short N-terminal 3_10_-helix and followed by a C-terminal α-helix. They frequently have one or more binding pocket(s) for small molecule ligand(s) [45], [46]. Inspection of the primary amino acid sequence of Japanin indicated that it is a lipocalin, a conclusion supported by comparisons with other tick-derived lipocalins (figure 6). Japanin conserves residue properties at the key positions described by Adam and colleagues [47] to a similar extent as tick proteins with resolved lipocalin structures (figure 6a), and shares the position of cysteine residues and the presence of a conserved motif ((Y/C)-hydrophilic-(L/M)-W-hydrophobic) with these and with other tick proteins thought to be lipocalins (figure 6b). This provisional description of Japanin as a lipocalin has recently been confirmed by the resolution of its crystal structure, details of which are currently being prepared for publication (personal communication, Susan Lea).

### Metastriate ticks produce a family of Japanin-like molecules with DC modulatory activity

**Figure 7:**
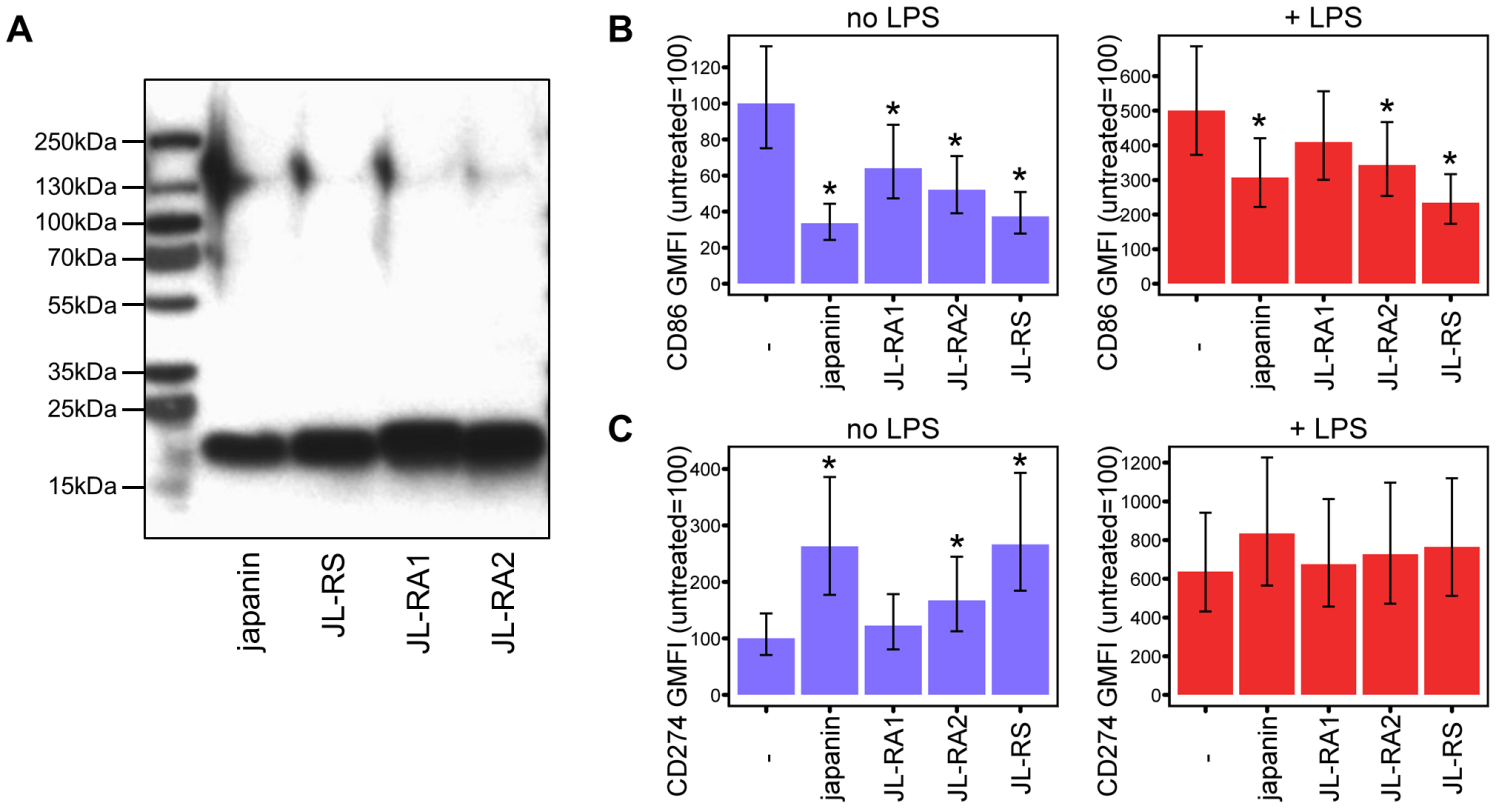
Japanin homologues modulate DC maturation. (A) Three Japanin homologues were successfully expressed in Sf9 cells, as shown by Western blotting with an anti-His tag antibody. ∼10 ng protein was loaded per lane. (B) & (C) Dendritic cells were cultured in the presence or absence of Japanin or Japanin homologues (500 ng/ml) and LPS (100 ng/ml) for 18–20 hours. CD86 (B) and CD274 (C) were then assessed by flow cytometry. Modelled means ±95% confidence intervals using data from three (cells with LPS) or four (cells without LPS) experiments are shown. * p<0.05, as compared to cells without Japanin or a Japanin homologue.

Tick genomes frequently encode several isoforms or homologues of a protein, apparently as a result of frequent gene duplication events during tick evolution [48]. We therefore investigated whether *R. appendiculatus* expresses any Japanin homologues, extending our studies to the closely related *Rhipicephalus sanguineus* species. We designed degenerate primers using the Japanin coding sequence, and used these to clone three sequences with similarity to Japanin: Japanin like-RS (JL-RS) from *R. sanguineus*, and Japanin like-RA1 and -RA2 (JL-RA1 and JL-RA2) from *R. appendiculatus* (Genbank accessions KC412663, KC412664, and KC412665; see materials and methods for cloning procedure). Each encodes a 175–177 residue peptide, comprising a 24 residue signal sequence (according to SignalP prediction) and a 151–153 residue mature peptide. Alignment of the predicted mature peptides with Japanin shows that JL-RS is 82% identical and 91% similar to Japanin, JL-RA1 is 50% identical and 74% similar, and JL-RA2 is 54% identical and 76% similar; similarity was calculated using a PAM250 matrix. The high levels of sequence homology between Japanin and the above homologues suggested shared function. To investigate this, we transfected HEK293T cells with pCDNA3.1 expression constructs encoding either polyhistidine-tagged Japanin, JL-RS, -RA1 or -RA2. The presence of recombinant protein within the supernatants was confirmed using anti-polyhistidine Western blot (figure 7a), and the transfectant supernatants were then assessed for their ability to modulate DC maturation, both spontaneously and in response to LPS. Each of the three homologues was found to modulate DC maturation overall (p<0.05), albeit with some individual differences in the extent of effect on each of these responses, and on CD86 vs. CD274 expression (figures 7b,7c). This suggests that their sequence similarity reflects a shared DC-modulatory function; we are currently evaluating whether or not other modulatory effects are similar to those of Japanin.

**Figure 8:**
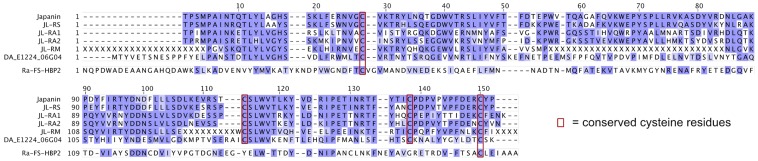
Sequence alignment of Japanin and its homologues. Alignments were generated with ClustalX and manually refined. Shading intensity indicates BLOSUM62 score. N-glycosylation sequences and conserved cysteine residues are boxed. Ra-FS-HBP2 (PDB ID 1QFT) is aligned as an example of a tick lipocalin with low sequence similarity to Japanin.

**Figure 9:**
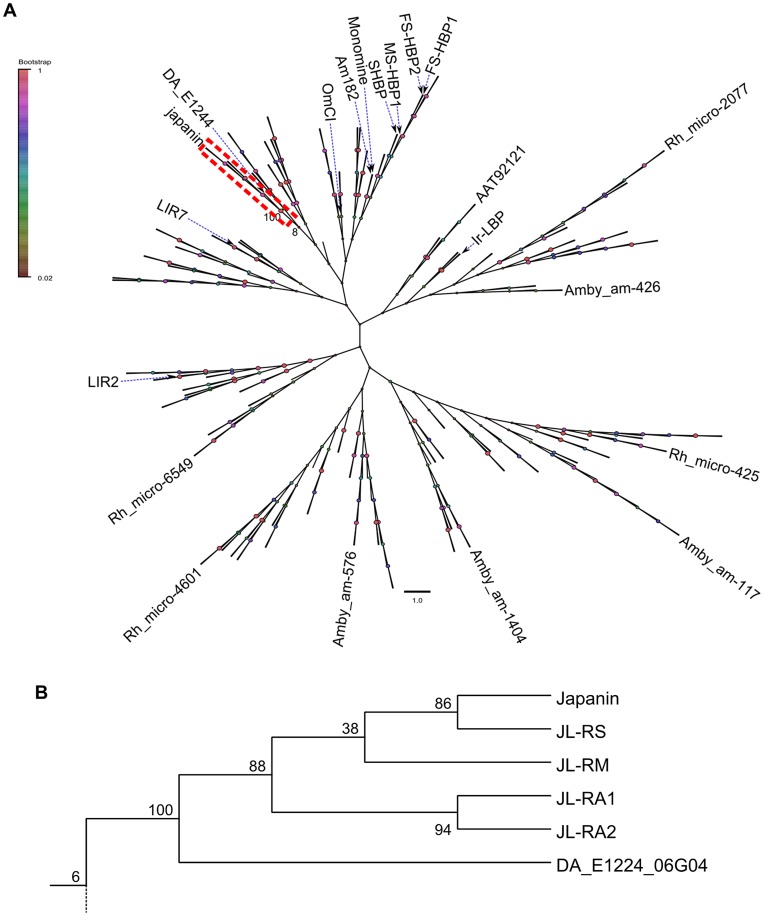
Japanin-like proteins form a clade within hard tick lipocalins. (A) A phylogenetic tree derived from maximum-likelihood analysis of hard tick lipocalins (including Japanin and its homologues), as well as the soft tick lipocalins OmCI, monomine and Am182. Sequences were aligned using ClustalX and manually refined, then Mega5.1 was used to construct a phylogeny. The frequency with which associated taxa clustered together in the bootstrap test is shown. For reasons of clarity, only selected protein names and bootstrap frequency labels are shown, and the Japanin clade is shown in detail in (B), with the full tree supplied as supplementary data.

Finally, in order to search for further Japanin-related sequences in public databases, tblastn searches were performed against the mature peptide sequence of Japanin (see materials and methods). The NCBI est database returned a cDNA derived from the metastriate tick *Dermacentor andersoni* with homology [32% identical, 53% similar] to Japanin (Genbank accession EG363153.1, labelled as DA_E1224_06G04 in figures 8 and 9). Furthermore, the Whole-genome shotgun contig database returned regions of *Rhipicephalus (Boophilus) microplus* genomic DNA whose translations are highly similar to Japanin [40–58% identical, 55–74% similar] (Genbank accessions ADMZ01222530.1; ADMZ01123695.1; ADMZ01066468.1; ADMZ01299354.1). All the identified homologues conserve Japanin's cysteine residues, which may play a structural role through disulphide bond formation (figure 8).

Tick lipocalins have conserved intron positions and phase [49]; this means that the identification of intron boundaries may be guided by intron-exon structure in addition to the more usual prediction of likely splicing site sequences [50]–[52]. This allowed us to predict intron boundaries with greater confidence than would otherwise be possible, and enables us to tentatively ascribe the three sequences with greatest similarity to Japanin (ADMZ01222530.1, ADMZ01123695.1, ADMZ01066468.1) to exons 2, 4 and 5 of a single gene (figure S7), which we provisionally entitle Japanin-like RM (JL-RM). The fourth sequence (ADMZ01299354.1), had the lowest similarity to Japanin, and overlaps with one of the other sequences. It may represent part of a second *R. microplus* Japanin homologue. Of course, in the absence of mRNA/cDNA sequences, it is possible that the three “JL-RM” sequences actually comprise parts of two or three distinct Japanin homologues. Further studies will be needed to determine whether these *D. andersoni* and *R. microplus* homologues also have DC-modulatory activity.

It is noteworthy that despite the availability of extensive genome and transcriptome data from prostriate ticks [53], clear examples of Japanin-related molecules were identified only from metastriates, suggesting that Japanin and its homologues are unique to metastriates.

### Japanin and its homologues represent a novel clade of lipocalins from metastriate ticks

The conserved number and positioning of cysteine residues, the conservation of key motifs, and the sequence homology to Japanin, allow us confidently to describe all of the above Japanin homologues as tick lipocalins. In order to estimate their evolutionary relationship to Japanin and to other tick lipocalins, we performed phylogenetic analysis, building a distance dendrogram using maximum-likelihood methods (see materials and methods). We compared the sequences of Japanin and its five identified homologues to 236 complete sequences derived from hard ticks, as well as 3 soft tick proteins with resolved structures. This analysis clearly shows that these molecules form a distinct clade within hard tick lipocalins, grouping in complete isolation from any previously identified proteins or putative proteins and with strong boot-strap support (figure 9). Note that for reasons of clarity, only selected proteins are named in figure 9, and the full tree is provided in Newick (.nwk) format as supplementary data in dataset S1.

## Discussion

Manipulation of dendritic cell function is a survival strategy adopted by a wide range of pathogens, from viruses and bacteria to protozoan and metazoan parasites [54]. Here we describe a novel tick-derived protein, Japanin, which combines the ability to extensively reprogramme DC maturation with a profound inhibitory effect on DC differentiation. Japanin appears to be one member of a unique family of highly specific DC-targeting proteins that seem to be produced only by metastriate ixodid ticks.

To our knowledge, previously described molecules derived from blood-feeding arthropods are either highly promiscuous in their cellular and molecular targets, or have limited effects on DC. For example two proteins, Maxadilan and LJM111, have been identified in the saliva of *Lutzomyia* sand flies that can alter the balance of cytokine secretion and costimulatory molecules by DC. The former apparently acts to favour the development of a Th2 response [55] although it was initially characterised as an exceptionally potent vasodilator [56] and is now known to have a widely-expressed receptor (PAC_1_) [57]; it is currently unclear whether the activity of LJM111 is in any way DC-specific [58], [59]. Sialostatin L, from *Ixodes scalpularis* ticks, alters DC cytokine secretion and costimulatory molecule expression in response to LPS [24], but it also alters T cell polarisation in the absence of DC [24], and inhibits proliferation of a T cell line [60]. As well as inhibiting cathepsin S, which plays a key role in MHC Class II molecule processing, and hence in antigen presentation [61], Sialostatin L also inhibits cathepsin L1, and so may play a role in limiting neutrophil activity (given the role of cathepsin L1 in IL-8 activation [62]) and/or in controlling tissue remodelling [63]. Salp15 (with its homologues), from *Ixodes* spp. ticks, is the only unambiguous example of a substantially DC-specific modulatory protein from a blood-feeding ectoparasite [27] but even this molecule also acts directly on CD4+ T cells [28]. Moreover, the effects of Salp15 on DC appear limited to a reduction in the secretion of certain pro-inflammatory cytokines by DC; unlike Japanin, it has no effect on membrane molecule expression or anti-inflammatory cytokine secretion.

Rather than simply inhibiting DC maturation, Japanin appears to hijack the normal maturation process and to redirect it in a totally different direction. It blocks LPS-induced secretion of pro-inflammatory and Th17- and Th1-promoting cytokines, and reduces expression of a key co-stimulatory molecule (CD86) required for T cell activation. Meanwhile, it also promotes secretion of the anti-inflammatory cytokine IL-10 and increases expression of CD274 (PD-L1), both of which are involved in the suppression of T cell immunity and the induction of antigen-specific tolerance [64]–[66]. Moreover, Japanin appears to modulate DC maturation in response to multiple “danger” signals. We have studied responses to bacterial LPS, a TLR4 agonist, in most detail, but its modulatory effects appear to extend to responses stimulated by TLR3 agonists (the natural ligand for which is viral double-stranded RNA), and by interferons, which are produced in response to tissue damage and infections. To our knowledge, no molecule has been previously reported to combine such a wide spectrum of potent and specific effects on DC maturation with the ability to modulate responses to a wide range of stimuli.

The ability of Japanin to modulate DC responses to a broad range of stimuli makes sense given that ixodid ticks might otherwise trigger DC maturation and T cell responses in a number of ways: (i) during attachment they cause tissue damage at the skin feeding site; and (ii) their saliva carries tick-borne pathogens. Hence tick feeding is likely to provide both endogenous (i.e. tissue damage-related) and exogenous (for example through TLRs) triggers for DC activation. Moreover, (iii) their saliva contains multiple bioactive proteins and peptides that help blood-feeding but which could potentially be recognised as foreign antigens by the host. Presumably to subvert such defences, prostriate (*Ixodes* spp.) ticks elaborate Salp15-like proteins which modulate both DC and T cell functions. The current study shows that metastriate ticks produce Japanin-like molecules which appear to modulate DC in a highly specific manner. We have been unable to detect binding of Japanin to T cells or B cells, or to any other major cell population in human blood, although we have not excluded a modulatory effect on macrophages, as reported recently for unfractionated *Rhipicephalus* (*B.*) *microplus* saliva [67]. In principle, Japanin may act directly on tissue-resident DC at the bite site and, being a comparatively small (∼20 kDa) molecule, it may also be carried in the lymphatics to influence lymph node-resident DC. Furthermore, the capacity of Japanin to modulate the differentiation of monocytes into DC in culture suggests that, *in vivo*, it may also act locally (and perhaps even within regional lymphoid tissues) to subvert the development of DC from their precursors.

Although the Japanin family of DC modulators appears to be restricted to metastriate tick genera, such as *Rhipicephalus* and *Dermacentor*, the lipocalin structure of Japanin is widely represented in the salivary gland transcriptome of blood-feeding arthropods [68], [69]. Lipocalins are found throughout the plant and animal kingdoms as well as bacteria, reflecting the robust and versatile nature of their β-barrelled structure. Typically, they are extracellular proteins that transport small hydrophobic ligands, although there are notable exceptions such as the tick lipocalins that bind small hydrophilic ligands [4], [47]. Examples of tick lipocalins that subvert host defences are the histamine- and complement-binding proteins [40], [48], and several mammalian lipocalins (dubbed “immunocalins”) have modulatory effects in immunity [70]. Japanin and its homologues appear to be the first examples of the lipocalin molecular architecture being employed to target DC.

In haematophagous ectoparasites, DC modulators have presumably evolved to suppress host immunity in order to facilitate blood-feeding. For those species that are vectors of pathogens, such molecules could also create a permissive environment for pathogen transmission. Although saliva and SGE from several species of mosquitoes, sand flies and ticks has been shown to both affect DC activity and enhance pathogen transmission, the relationship between DC modulation and pathogen transmission has not been resolved [71]–[73]. For example, Salp15 facilitates transmission of the Lyme disease spirochete from the tick vector to the host [74]. However, it is unclear whether this is due to the binding of Salp15 to: (i) DC-SIGN on DCs, thus inhibiting the spirochete-induced production of pro-inflammatory cytokines by DCs and so modulating DC-induced T cell activation [27]; (ii) CD4, thereby inhibiting T cell activation [28], [75], [76]; and/or (iii) OspC, an outer surface protein on the spirochete, hence protecting the spirochete from antibody-mediated killing [77]. Likewise, Maxadilan promotes transmission of *Leishmania* parasites although the relative contribution of DC modulation to enhanced transmission is unresolved [78].

Metastriate ticks are important vectors of human and animal pathogens, so could Japanin facilitate tick-borne transmission? *In vivo* experimental studies showed that an unidentified protein in SGE of *R. appendiculatus* promotes transmission of Thogoto virus and tick-borne encephalitis virus (TBE virus), and that TBE virus infects Langerhans cells [72], [79]. The effect of saliva components on *Theileria parva*, the cause of the devastating African cattle disease, East Coast fever, is unknown. Interestingly, tick-borne transmission of this protozoan pathogen commences about 3 days after initiation of *R. appendiculatus* feeding, coinciding with the production of Japanin [80]. The existence of a Japanin homologue in *Dermacentor andersonii*, a major vector of Rocky Mountain spotted fever, is also of note. Further studies are needed to determine whether Japanin and its homologues play a role in the transmission of tick-borne pathogens; one possible approach would be through their RNA-mediated knockdown [81].

Our findings describe an entirely new and highly specific class of DC modulators, potentially providing a novel mechanism for the control of adaptive immunity. We anticipate that further work will reveal the mechanism by which Japanin exerts its effects on DC, besides revealing its effects on development of T cell responses and the adaptive response as a whole. Ultimately, these DC modulators in saliva of metastriate ticks may help enable ectoparasites to feed successfully on their hosts without provoking effective immune responses, while at the same time creating a permissive environment for pathogen transmission to their hosts.

## Materials and Methods

### Reagents

Lipopolysaccharide (LPS) was from *E. coli* 055:B5, and purchased from Sigma (catalogue #L4005). Polyinosinic:polycytidylic acid (poly I:C) and CL097 were from Invivogen. Human IFNα2, IFNγ, TNFα, IL-4 and soluble CD40 ligand were from Peprotech. Human GM-CSF was from Gentaur. Recombinant OmCI was produced as previously described [40]. Foetal calf serum (FCS) was from Invitrogen. Plasmids were from Invitrogen (pCR2.1-TA, pCR-Blunt II-TOPO & pCDNA3.1), Clontech (pBacPAK8) and Novagen (pET52b). Unless otherwise noted, PCR was carried out using Phusion Hot Start DNA polymerase (NEB). Phosphate buffered saline (PBS) and Hanks Balanced Salt Solution were from PAA.

### Cell culture

Mammalian cells were cultured at 37°C/5% CO_2_ in complete RPMI (C-RPMI), consisting of RPMI 1640 (PAA) supplemented with 10% FCS, 100 U/ml penicillin (PAA), 100 µg/ml streptomycin (PAA). Tissue culture plastics were from Corning. Sf9 insect cells were cultured at 28°C in Sf900III serum-free medium (Invitrogen). Sf9 liquid culture was in Erlenmeyer flasks with shaking at 110 rpm. Standard *E. coli* strains and techniques were used to produce plasmids during molecular cloning. Human blood products were from anonymous healthy donors, and supplied by the National Blood Service (England & Wales).

### Generation of human dendritic cells

Human monocyte-derived DC were generated using a protocol derived from the method of Sallusto and Lanzavecchia [82]. Peripheral blood mononuclear cells (PBMC) were isolated from Buffy coats and leucocyte cones using gradient centrifugation with Lymphoprep (Axis Shield). Monocytes were isolated from PBMC by negative selection using the EasySep Human Monocyte Enrichment Kit (Stemcell) as per manufacturer's instructions, then cultured at 5×10^5^/ml in C-RPMI supplemented with 1000 U/ml human GM-CSF and 100 ng/ml human IL-4. Cultures were fed after three days by replacing one third of the medium with fresh C-RPMI supplemented with 3000 U/ml GM-CSF and 300 ng/ml IL-4, and cells were harvested for use in assays after 5 or 6 days of culture. Prior to some assays, DC were frozen in Voluven (Fresenius Kabi) supplemented with DMSO (Hybrimax grade, Sigma) and FCS to give final concentrations of 5.5% hydroxyethyl starch 130/0.4, 4.8% DMSO and 3.8% FCS in isotonic saline. Freezing was carried out at 1°C/minute.

### DC activity assay

DC were cultured at 1×10^6^ cells/ml in flat-bottomed 96-well tissue culture plates in C-RPMI supplemented with 1000 U/ml GM-CSF and 100 ng/ml IL-4. Japanin was added to 500 ng/ml (unless otherwise stated), and a maturation stimuli was either added immediately or after 24 hours in culture. Cells were then cultured for 18–22 hours, and analysed by flow cytometry. In some experiments, multiplex measurement of supernatant cytokine concentrations was also performed. In the experiments shown in figures S1 and S2, sufficient SGE was added to give a final SGE-derived protein concentration of 50 µg/ml

### T cell isolation and stimulation

PBMC were obtained from leucocyte cones as described above, and T cells were isolated by negative selection using the Easysep human T cell enrichment kit (Stemcell) as per manufacturer's instructions. They were then stimulated by culture with Human T-activator CD3/CD28 Dynabeads (Life Technologies) for four days, as per manufacturer's instructions.

### Tick rearing and salivary gland extract preparation

*R. appendiculatus* ticks were reared according to Jones *et al.* [83] Salivary glands were dissected under a microscope and rinsed briefly in cold PBS. Salivary gland extract (SGE) was prepared by disruption of freshly-prepared salivary glands in PBS with a 1 ml Dounce homogenizer. The SGE was clarified by centrifugation (>10000 g for 3 min) and stored at −20°C.

### SGE fractionation

SGE from 350 salivary glands was diluted in 50 mM Na_2_HPO_4_/50 mM NaCl (pH7.0) and passed through a 1 ml Hi-Trap Q sepharose anion exchange column (GE). Unbound material (Q column flowthrough) was concentrated to a final volume of 500 µl using a 5000MWCO Vivaspin 6 centrifugal concentrator (GE Healthcare) which had been pre-treated with γ-globulin to prevent non-specific absorbance of proteins. The Q column flowthrough was then fractionated by gel filtration over a Superdex 75 HR10/30 column (GE Healthcare) using 50 mM Hepes (pH7.6), 150 mM NaCl as running buffer, and each fraction assayed for DC modulatory activity. Consecutive active fractions were pooled, dialysed against 50 mM HEPES (pH 8.3), and fractionated by High Performance Liquid Chromatography (HPLC) on a C4 column, with elution using a 0–100% gradient of acetonitrile. HPLC fractions were freeze-dried under vacuum, redissolved in PBS, and assayed for DC modulatory activity. The fraction with maximal activity was used for Edman degradation sequencing.

### Japanin cloning

The template for Japanin cloning was cDNA generated from 1 day-fed female *R. appendiculatus* salivary glands. RNA was isolated from 30 salivary glands using Trizol reagent (Invitrogen), and cDNA generated using ImPromII reverse transcriptase (Promega). Initial cloning of Japanin sequence was performed using *Taq* DNA polymerase (NEB) in nested PCR with degenerate primers designed against the N-terminal peptide sequence. A ∼600 bp product was gel purified using the QIAquick gel extraction kit (Qiagen) and ligated into the pCR2.1-TA cloning vector.) Sequencing of this construct revealed the 3′ region of the Japanin coding sequence; this was used to design primers for the amplification of the 5′ region using 5′ RACE System for Rapid Amplification of cDNA Ends (Invitrogen) in conjunction with Japanin-specific primers. Amplified DNA was gel purified and sequenced, providing the 5′ region of the coding sequence. The 5′ and 3′ sequences obtained thus far were then used to design primers for the amplification of full-length Japanin coding sequence using two rounds nested PCR, with the second round using primers which added a 5′ BamHI restriction site and a 3′ NotI restriction site. The second round product was digested with BamHI and NotI (NEB), and ligated into similarly digested pBacPAK8 to generate pBacPAK8-Japanin. In order to obtain a polyhistidine-tagged version of Japanin, nested PCR was performed with Phusion DNA polymerase using pBacPAK8-Japanin as a template, employing reverse primers designed so as to replace the 3′ stop sequence and NotI-site with DNA encoding two glycine residues (to serve as a flexible linker) and six histidine residues (the “polyhistidine tag”), followed by a stop sequence, and then finally a NotI site. The product from this PCR was digested with BamHI and NotI, and ligated into similarly digested pBacPAK8 (to produce pBacPAK8-Japanin-his), pCDNA3.1 (pCDNA3.1-Japanin-his) and pET52b (pET52b-Japanin-his).

### Homologue cloning

Partial sequences of JL-RA1, JL-RA2 and JL-RS were obtained from Rhipicephalus appendiculatus (JL-RA1/2) or R. sanguineus (JL-RS) cDNA expression libraries which had been previously generated in the Lambda Zap II vector (Stratagene). PCR was performed using a degenerate, Japanin-derived forward primer (ACMSAKACYCTYTACCTYGYG) in combination with either a vector specific reverse primer (TTATGCTGAGTGATACCC), in the case of JL-RA2, or a Japanin-specific reverse primer (ATATGCGGCCGCTTATGGATAGCACCTCTCGT), in the case of JL-RA1 and -RS. PCR products were cloned into pCR-Blunt II-TOPO and sequenced, providing sequences for the 3′ region of each DNA. Sequences of the 5′ region of each were then obtained from the same libraries by PCR using a vector-specific forward primer (CGCAATTAACCCTCACTAAAGGGAAC) with gene-specific reverse primers (CGTTAGTTTCAGTGAACGTGAGTGTCC for JL-RA1; CGTTTGGTATCTTCATTTTAGATGAGTATCC for JL-RA2; CATGAGAACAGCTTCGATGAATATGC for JL-RS), and products cloned into pCR-Blunt II-TOPO and sequenced. Full-length cDNAs, each with the addition of sequence encoding a C-terminal diglycine linker and a polyhistidine tag (GGHHHHHH) were obtained as synthetic genes (from DNA2.0) and subcloned into pBacPAK8 using standard techniques. Recombinant JL-RA1, -RA2 and -RS was produced and purified as described below.

### Proteinase K treatment

Proteinase K treatment of SGE-3F was performed by incubation with 150 µg/ml Proteinase K (Sigma) for 2 hours at 50°C, followed by heating to 98°C for 10 minutes to inactivate the enzyme.

### Protein expression

Recombinant baculovirus was obtained using the approach of Possee *et al.* [84] Briefly, Sf9 cell monolayer was co-transfected with flashBac Gold baculovirus (Oxford Expression) and pBacPAK8 transfer vector (described above), using Cellfectin (Invitrogen) as per manufacturer's instructions. Recombinant virus was amplified by infection of Sf9 cells in liquid culture at a low multiplicity-of-infection (moi), and the amplified virus used to infect Sf9 liquid cultures at moi = 2 for protein expression. Viral titre was assessed by plaque assay.

### Protein purification

The medium was cleared by centrifugation (2000 g, 10 min) 72 hours after infection, and proteins were precipitated by adding polyethylene glycol (PEG4000, Sigma; 18 g/100 ml). The precipitate was dissolved in HBSS (pH 7.4), loaded on to a 1 ml Talon column (Clontech), and eluted using 150 mM imidazole. The protein-containing eluate fractions were pooled, concentrated using a 9K MWCO Pierce Protein Concentrator (Thermo Scientific), then further purified by size exclusion chromatography with a Superdex 75 HR10/30 column (GE Healthcare) using PBS (pH7.4) as running buffer. Concentration of purified protein was measured by its absorbance at 280 nm using extinction coefficients reported by the ProtParam tool (http://web.expasy.org/protparam/). Purity was confirmed by silver stain of SDS-PAGE gels.

### Western blotting

SDS-PAGE was performed using precast Precise Tris-HEPES gels (Thermo Scientific) as per manufacturer's instructions. Proteins were wet transferred to PVDF membrane (Thermo Scientific) using 30 V for 1 hour in Towbin buffer (25 mM Tris, 192 mM glycine) with 20% methanol. Membranes were blocked with StartingBlock T20-PBS (Thermo Scientific), then stained first with biotinylated anti-His tag antibody (Penta-His, Qiagen), and then with streptavidin-HRP (Jackson ImmunoResearch). All staining steps, and extensive washing, was in PBS/0.05% Tween 20. Bands were visualised by luminescent substrate (ECL, Thermo Scientific) with X-ray film (CL-XPosure, Thermo Scientific).

### Flow cytometry

Cells were stained in PBS/2% FCS and analysed with a FACSCanto flow cytometer (Becton Dickinson). The following antibodies were used: 5C3 (anti-CD40, APC-conjugated); HB15e (anti-CD83, FITC-conjugated); GL1 (anti-CD86, PE-conjugated); MIH1 (anti-CD274, PE-Cy7-conjugated); LN3 (anti-HLA-DR, APC-conjugated). All were from eBioscience. Isotype control antibodies showed negligible binding to DC. Cells were gated according to FSC/SSC, and, in some experiments, according to exclusion of 7AAD (Sigma). Japanin did not increase the frequency of 7AAD-staining cells.

### Multiplex analysis of culture supernatants

Clarified tissue culture supernatants were diluted with 1 volume of PBS and stored at −20°C. They were analysed using Milliplex MAP Luminex beads (Millipore) as per manufacturer's instructions.

### Binding assays

Recombinant Japanin and OmCI were labelled with DyLight 649 using DyLight 649 Amine-Reactive Dye (Thermo Scientific) as per manufacturer's instructions. In the experiment shown in figure S6, examining the binding of Japanin at different points during the differentiation of DC from monocytes, proteins were instead labelled with Alexa Fluor 488, using the Alexa Fluor 488 Microscale Protein Labelling Kit (Life Technologies) as per manufacturer's instructions. Cells were incubated with 100 ng/ml labelled Japanin or 340 ng/ml labelled OmCI for 1 hour on ice in HBSS (containing 1.3 mM Ca^2+^ 0.8 mM Mg^2+^)/2% FCS, washed extensively, and analysed by flow cytometry.

For the determination of Japanin binding to PBMC subsets, PBMC were incubated with Fc block (Miltenyi Biotec) for 15 minutes on ice, washed, then incubated with DyLight 649-labelled Japanin or OmCI as described above, but with the addition of the following antibodies: anti-CD1c-Brilliant Violet 421 (clone L161, Biolegend); CD3-biotin (clone OKT3, Biolegend); CD7-biotin (clone 124-1D1, eBioscience); CD14-Brilliant Violet 650 (clone M5E2, Biolegend); CD11c-PE-Texas Red (clone B-ly6, BD Biosciences); CD19-biotin (clone HIB19, eBioscience); CD20-biotin (clone2H7, eBioscience); CD45-eFluor 605NC (clone HI30, eBioscience); CD56-biotin (clone HCD56, Biolegend); CD123-PerCP-Cy5.5 (clone6H6, Biolegend); CD141-PE (clone AD5-14H12, Miltenyi Biotec); HLA-DR-V500 (clone G46-6, BD Biosciences). The cells were washed, then incubated with streptavidin-Alexa Fluor 700 (Life Technologies), and washed again prior to analysis. Dead cells were excluded by using Fixable Viability Dye eFluor 780 (eBioscience) in the first staining step. The biotinylated antibody panel visualised with streptavidin-Alexa Fluor 700 (CD3/CD7/CD19/CD20/CD56) is referred to in the text and figures as “lineage” (or “lin”).

### Database searches

Translated BLAST (Basic Local Alignment Search Tool [85] searches were performed with the mature Japanin peptide sequence as the query, using the NCBI online interface (http://blast.ncbi.nlm.nih.gov/). Similarity scores were obtained with blastp or tblastn, as appropriate, using the same interface, and with a PAM250 matrix.

### Phylogenetic analysis

An initial group of 4 tick lipocalins with published structures (FS-HBP2 [4], Am182 [86], Monomine [86] and OmCI [87]) were aligned using ClustalX [88], and this seed alignment used to construct a gap penalty mask. This mask was then employed in the alignment of an additional 242 hard tick lipocalins using ClustalX. Sequences for alignment were taken from: (i) the table provided as supplementary data by Francischetti and colleagues [89], from which all complete sequences identified as hard tick lipocalins, were used, with the exception of those described as group VIII, which we do not believe to be lipocalins (based on the absence of conserved sequence features); (ii) LIR2 and LIR7 [90]; (iii) Ir-LBP [91]; (iv) the sequences described in this paper. Sequences are named according to their published abbreviation, Genbank accession number, or as referred to by Francischetti and colleagues. This alignment was manually refined to align key conserved sequence features, and MUSCLE [92] used to realign subsections of the alignment between conserved features. The edges were trimmed manually to leave a conserved core. Evolutionary history was then inferred using the maximum-likelihood method. After model selection according to AICc and BIC criteria, the Whelan and Goldman + Freq. model [93] was used, with initial tree(s) for the heuristic search generated by applying the Neighbour-Joining method to a matrix of pairwise distances estimated using a JTT model. A discrete Gamma distribution was used to model evolutionary rate differences among sites (5 categories (+G, parameter = 7.3058)). The bootstrap consensus tree inferred from 50 replicates is taken to represent the evolutionary history of the taxa analysed. All positions with less than 90% site coverage were eliminated. That is, no more than 10% alignment gaps, missing data, or ambiguous bases were allowed at any position. There were a total of 112 positions in the final dataset. An alternative analysis where all positions with less than 95% site coverage were supported the conclusion that Japanin-like proteins form a clade, as did an analysis using the neighbour-joining method (with evolutionary distances computed using the JTT matrix-based method).

### Statistical analysis

For analysis of cytokine secretion and flow cytometry data, it was necessary to fit a two-level model in order to take into account within-donor correlations. Accordingly, a linear mixed effects model with donor as a random effect was employed, with p values estimated using Markov chain Monte Carlo sampling (MCMC). Normality and stability of variance were also required; this was achieved by means of a log transformation. The inverse (exponential) transformation to arrive at the model values involves Jensen's inequality bias: this is a 2^nd^ order effect which varies according to the reciprocal of the sample size, and in this case was negligible for practical purposes. In some cases, above-scale values necessitated putting data into ordered categories, after which a two-level ordinal regression model, with donor as a random effect, was fitted successfully.

### Software

Statistical analyses were performed with R [94], using the lme4 [95] and ordinal [96] packages for modelling, and the languageR package [97] for MCMC sampling. Line and bar charts were produced with R, using the ggplot2 [98], plotrix [99] and Cairo [100] packages. Flow cytometry data was collected using FACSDiva (BD Biosciences), and analysed and plotted with FlowJo (Tree Star). Sequence alignments were viewed and edited using UGENE [101], and formatted for publication with Jalview. Phylogenetic analyses were conducted using MEGA version 5.1 [102], and trees were formatted for publication with FigTree 1.4 (http://tree.bio.ed.ac.uk/software/figtree/).

### Accession numbers

UniProt accession numbers for proteins mentioned in the text can be found in Table S1.

## Supporting Information

Dataset S1 **A phylogenetic tree of hard tick lipocalins in detail.** Detail of the data presented in simplified form in figure 9. This file is in .nwk format and can be accessed through Mega5 software, which is provided free for research and education at http://www.megasoftware.net/mega.php (for Windows) or http://www.megasoftware.net/megamac.php (for Mac).
(NWK)

Figure S1 **Salivary gland extract from 3 day-fed female** ***Rhipicephalus appendiculatus*** **ticks modulates DC maturation.** Dendritic cells were incubated with 50 µg/ml salivary gland extract for 24 hours prior to the addition of LPS (100 ng/ml) for a further 18–20 hours. CD86 expression was then analysed by flow cytometry. Salivary gland extracts were generated from male (M) or female (F) ticks, either unfed (D0), or fed for three (D3) or six (D6) days.
(TIF)

Figure S2 **The DC-modulatory activity of salivary gland extract is abolished by treatment with Proteinase K.** Salivary gland extracts from three-day fed female *R. appendiculatus* ticks were treated with Proteinase K to digest salivary gland proteins. Dendritic cells were incubated with these, or mock-treated, extracts for 24 hours prior to the addition of LPS (100 ng/ml) for a further 18–20 hours. CD86 expression was then analysed by flow cytometry, and expressed as the product of the percentage of cells expressing CD86 cells and their geometric mean fluorescence intensity.
(TIF)

Figure S3 **Japanin does not bind to activated T cells.** Human T cells were stimulated for four days with (B) CD3/CD28 beads, or left untreated (A), then incubated on ice for 1 hour with 100 ng/ml Japanin-DyLight 649 (filled histograms) or 340 ng/ml OmCI (open histograms), and washed. Binding was assessed by flow cytometry.
(TIF)

Figure S4 **Gating strategies for differentiation of PBMC cell-types.** PBMC were (A) initially gated by forward-scatter & side-scatter, then (B) live leucocytes were selected by gating on CD45 & Viability stain. (C) Live leucocytes were subdivided according to CD14 & HLA-DR expression, and (D) the HLA-DR^+^CD14^−^ subset (antigen-presenting cells other than monocytes) was gated according to lineage & CD16. The CD16^−^lin^−^ subset was further subdivided into DC subsets as shown in figure 2.
(TIF)

Figure S5 **Additional data showing cytokine secretion in response to LPS with and without the presence of Japanin.** Dendritic cells were cultured in the presence or absence of Japanin (500 ng/ml) and LPS (100 ng/ml) for 18–20 hours. The concentration of the indicated cytokines and chemokine in the culture supernatant was then measured by Luminex. Modelled means ±95% confidence intervals using data from at least four experiments are shown. ** p<0.01, • p<0.1, NS p>0.05.
(TIF)

Figure S6 **The ability to bind Japanin is upregulated during the differentiation of monocytes into dendritic cells.** Freshly isolated monocytes, or those cultured with GM-CSF and IL-4 for 1–5 days, were incubated with 100 ng/ml Japanin-Alexa 488 (filled histograms) or 100 ng/ml OmCI-Alexa 488 (open histograms), incubated on ice for 1 hour, and washed. Binding was assessed by flow cytometry.
(TIF)

Figure S7 **Splicing site predictions suggest that three short** ***Rhipicephalus*** **(** ***Boophilus*** **)** ***microplus*** **genomic sequences may be three exons of a Japanin homologue.** (A) Translation of three *R. microplus* sequences obtained from the NCBI whole genome shotgun database. Putative splicing sites, with the same intron phase as the conserved lipocalin pattern, are in bold and underlined, with splice junctions marked by green stars. (B) Alignment of the three *R. microplus* sequences with Japanin, assuming splicing follows the suggested pattern. (C) Alignment of the three *R. microplus* sequences (“JL-RM”) with *R. appendiculatus* female-specific histamine binding protein 2 (Ra-FS-HBP2), a tick lipocalin with a known intron structure. Blue stars indicate the position of Ra-FS-HBP2 introns, while green stars show the position of introns according to the splicing sites indicated in A. Note that the phase of each putative JL-RM intron is the same as the closest Ra-FS-HBP2 intron.
(TIF)

Table S1 **Accession numbers of proteins.** UniProt accession numbers of proteins mentioned in the text, and not given elsewhere, are listed here.
(DOCX)
