# Supplementary figures and images for: Novel Immunomodulators from Hard Ticks Selectively Reprogramme Human Dendritic Cell Responses
Source: PLoS Pathog. 2013 Jun 27;9(6):e1003450. doi: 10.1371/journal.ppat.1003450 (PMC3695081; doi:10.1371/journal.ppat.1003450)

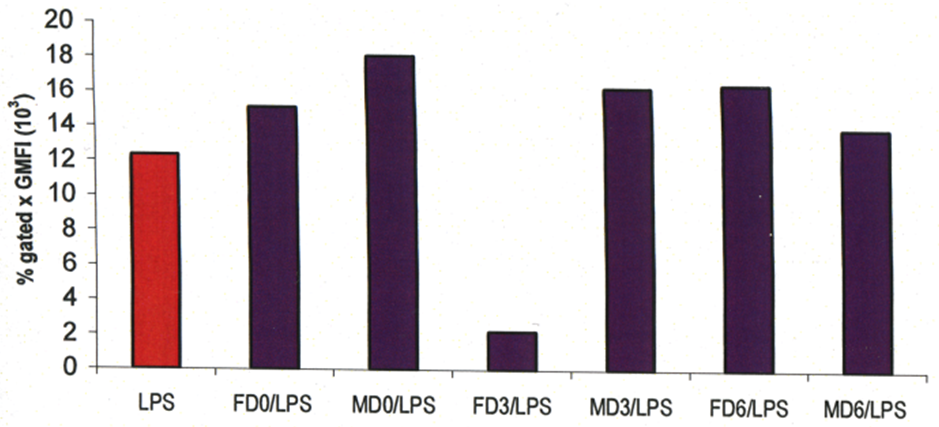

Supplement: Figure S1 — Salivary gland extract from 3 day-fed female Rhipicephalus appendiculatus ticks modulates DC maturation. Dendritic cells were incubated with 50 µg/ml salivary gland extract for 24 hours prior to the addition of LPS (100 ng/ml) for a further 18–20 hours. CD86 expression was then analysed by flow cytometry. Salivary gland extracts were generated from male (M) or female (F) ticks, either unfed (D0), or fed for three (D3) or six (D6) days. (TIF) [file ppat.1003450.s002.tif]

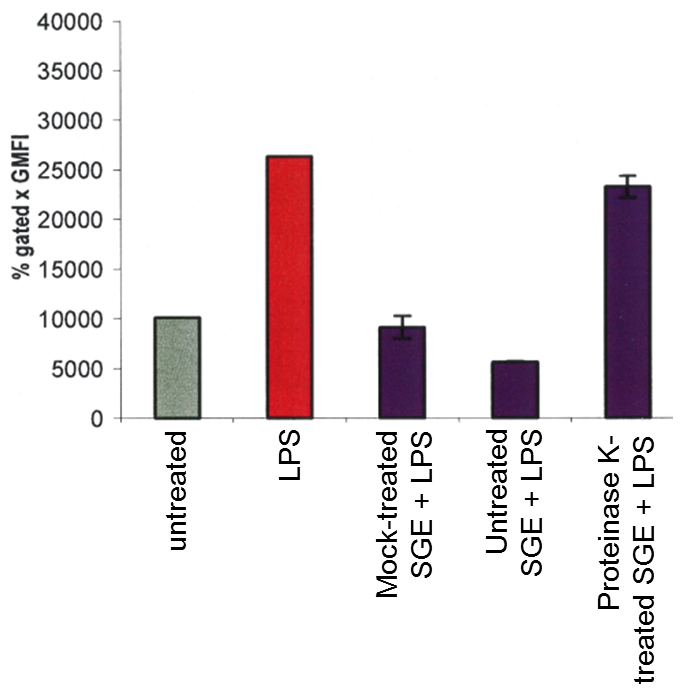

Supplement: Figure S2 — The DC-modulatory activity of salivary gland extract is abolished by treatment with Proteinase K. Salivary gland extracts from three-day fed female R. appendiculatus ticks were treated with Proteinase K to digest salivary gland proteins. Dendritic cells were incubated with these, or mock-treated, extracts for 24 hours prior to the addition of LPS (100 ng/ml) for a further 18–20 hours. CD86 expression was then analysed by flow cytometry, and expressed as the product of the percentage of cells expressing CD86 cells and their geometric mean fluorescence intensity. (TIF) [file ppat.1003450.s003.tif]

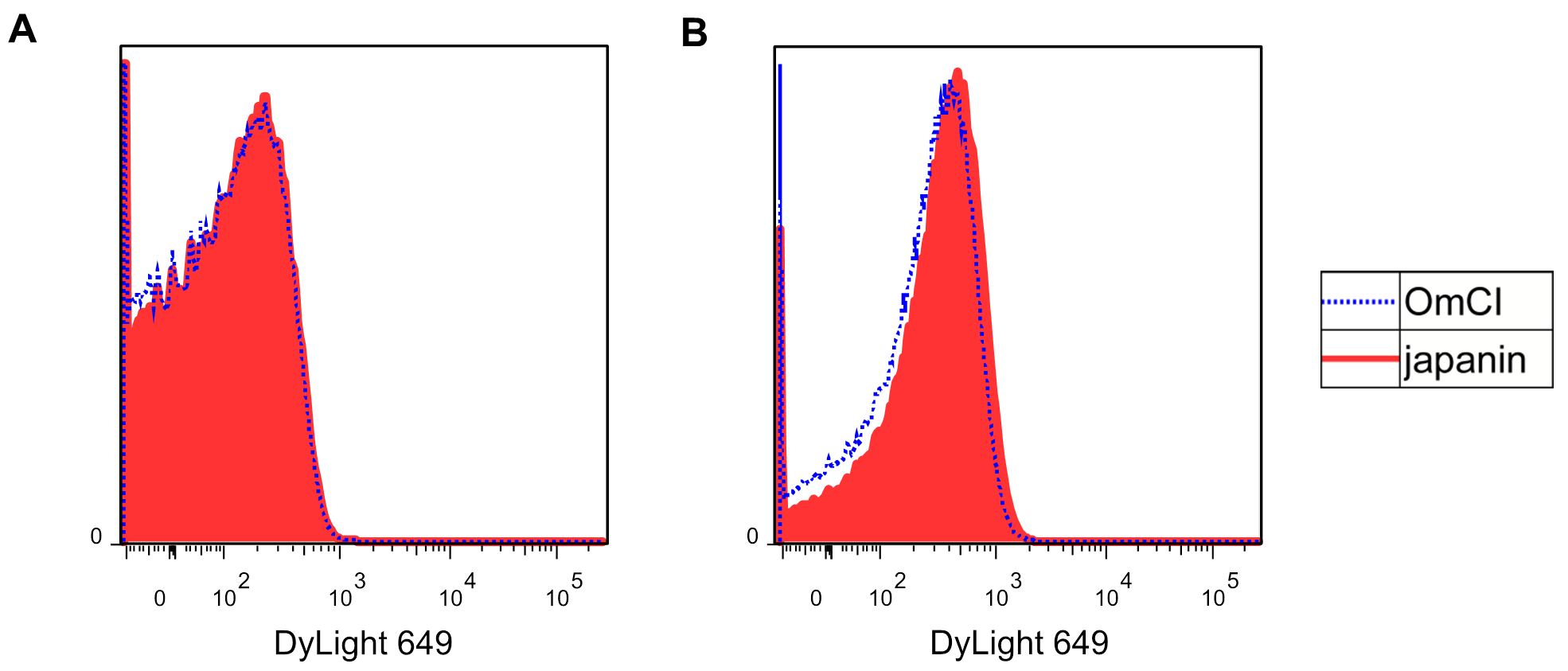

Supplement: Figure S3 — Japanin does not bind to activated T cells. Human T cells were stimulated for four days with (B) CD3/CD28 beads, or left untreated (A), then incubated on ice for 1 hour with 100 ng/ml Japanin-DyLight 649 (filled histograms) or 340 ng/ml OmCI (open histograms), and washed. Binding was assessed by flow cytometry. (TIF) [file ppat.1003450.s004.tif]

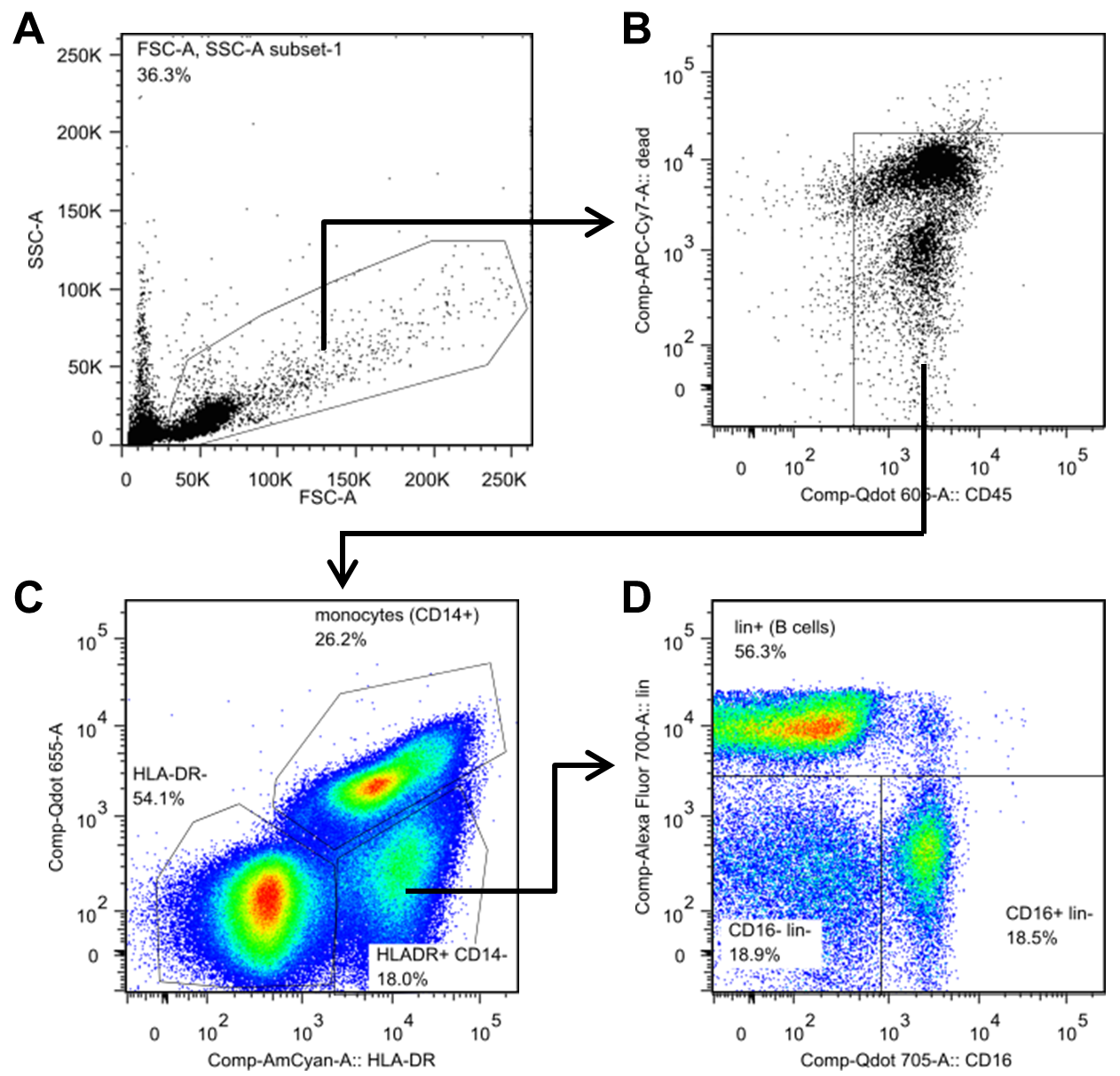

Supplement: Figure S4 — Gating strategies for differentiation of PBMC cell-types. PBMC were (A) initially gated by forward-scatter & side-scatter, then (B) live leucocytes were selected by gating on CD45 & Viability stain. (C) Live leucocytes were subdivided according to CD14 & HLA-DR expression, and (D) the HLA-DR+CD14− subset (antigen-presenting cells other than monocytes) was gated according to lineage & CD16. The CD16−lin− subset was further subdivided into DC subsets as shown in figure 2. (TIF) [file ppat.1003450.s005.tif]

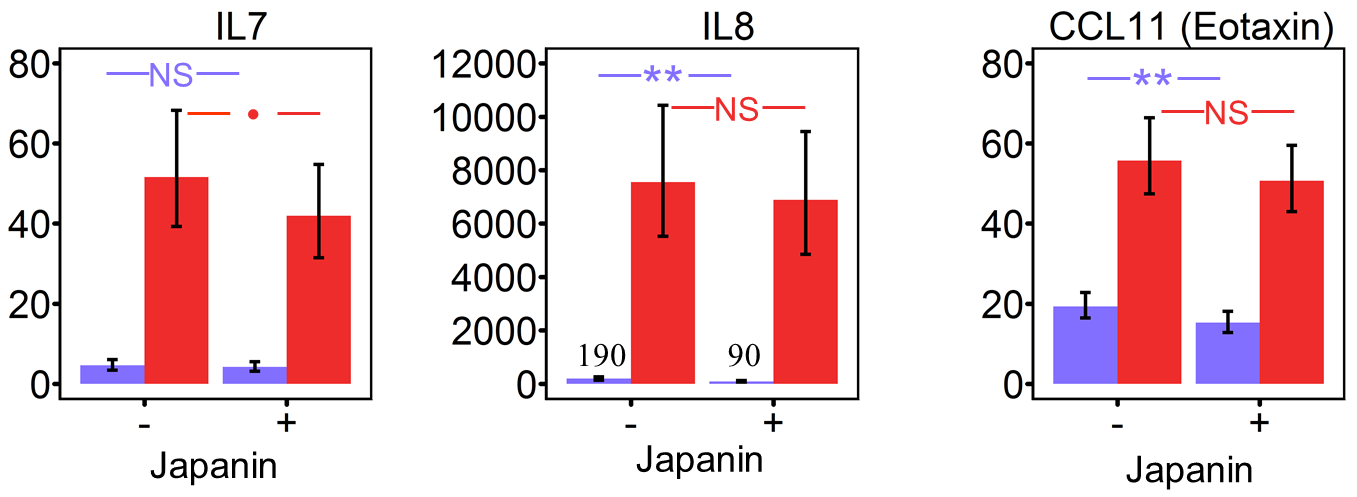

Supplement: Figure S5 — Additional data showing cytokine secretion in response to LPS with and without the presence of Japanin. Dendritic cells were cultured in the presence or absence of Japanin (500 ng/ml) and LPS (100 ng/ml) for 18–20 hours. The concentration of the indicated cytokines and chemokine in the culture supernatant was then measured by Luminex. Modelled means ±95% confidence intervals using data from at least four experiments are shown. ** p<0.01, • p<0.1, NS p>0.05. (TIF) [file ppat.1003450.s006.tif]

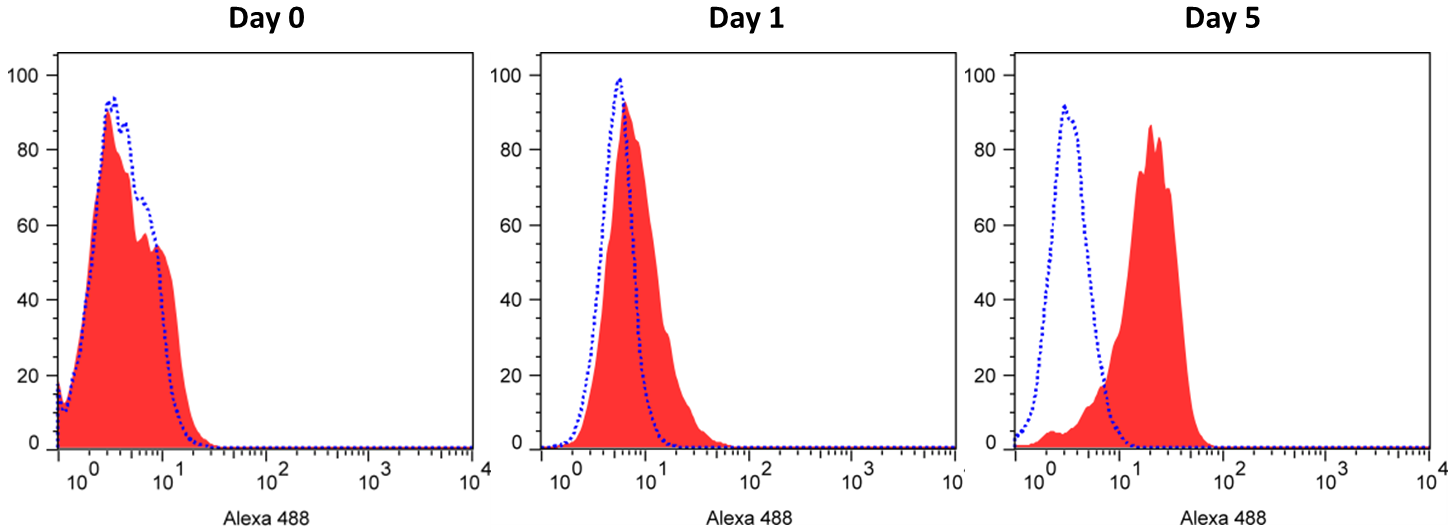

Supplement: Figure S6 — The ability to bind Japanin is upregulated during the differentiation of monocytes into dendritic cells. Freshly isolated monocytes, or those cultured with GM-CSF and IL-4 for 1–5 days, were incubated with 100 ng/ml Japanin-Alexa 488 (filled histograms) or 100 ng/ml OmCI-Alexa 488 (open histograms), incubated on ice for 1 hour, and washed. Binding was assessed by flow cytometry. (TIF) [file ppat.1003450.s007.tif]

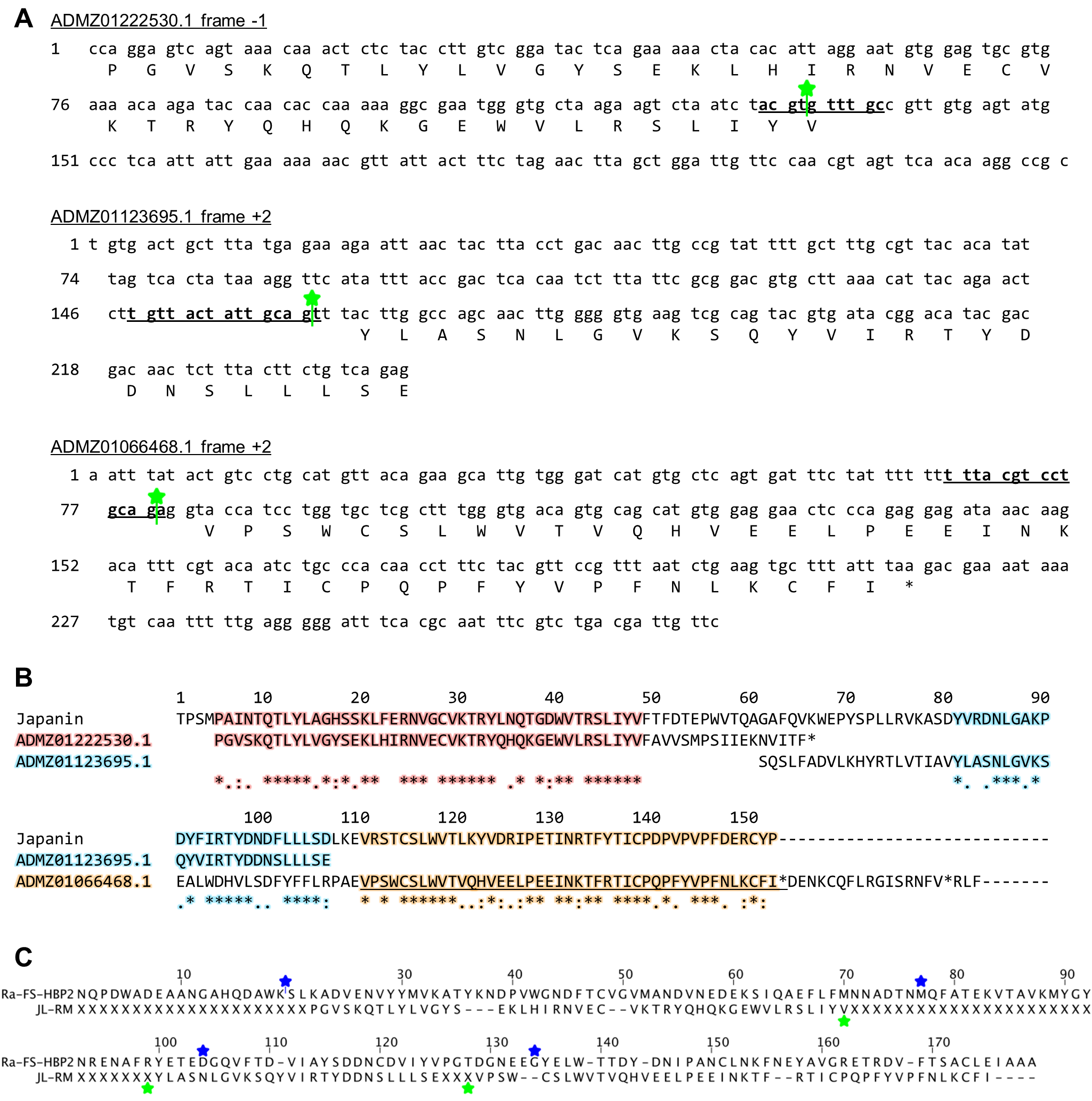

Supplement: Figure S7 — Splicing site predictions suggest that three short Rhipicephalus ( Boophilus ) microplus genomic sequences may be three exons of a Japanin homologue. (A) Translation of three R. microplus sequences obtained from the NCBI whole genome shotgun database. Putative splicing sites, with the same intron phase as the conserved lipocalin pattern, are in bold and underlined, with splice junctions marked by green stars. (B) Alignment of the three R. microplus sequences with Japanin, assuming splicing follows the suggested pattern. (C) Alignment of the three R. microplus sequences (“JL-RM”) with R. appendiculatus female-specific histamine binding protein 2 (Ra-FS-HBP2), a tick lipocalin with a known intron structure. Blue stars indicate the position of Ra-FS-HBP2 introns, while green stars show the position of introns according to the splicing sites indicated in A. Note that the phase of each putative JL-RM intron is the same as the closest Ra-FS-HBP2 intron. (TIF) [file ppat.1003450.s008.tif]
