# Supplementary material for: Novel Immunomodulators from Hard Ticks Selectively Reprogramme Human Dendritic Cell Responses
Source: PLoS Pathog. 2013 Jun 27;9(6):e1003450. doi: 10.1371/journal.ppat.1003450 (PMC3695081; doi:10.1371/journal.ppat.1003450)
Supplement: Table S1 — Accession numbers of proteins. UniProt accession numbers of proteins mentioned in the text, and not given elsewhere, are listed here. (DOCX) [file ppat.1003450.s009.docx]

Table S1. Accession numbers of proteins mentioned in the text.

| **Protein** | **UniProt ID** |
| --- | --- |
| AM182 | Q09JR9 |
| Cathepsin L1 | P07711 |
| Cathepsin S | P25774 |
| CCL11 (eotaxin) | P51671 |
| CD4 | P01730 |
| CD8 | P01732 + P10966 |
| CD11c | P20702 |
| CD123 | P26951 |
| CD14 | P08571 |
| CD141 | P07204 |
| CD16 | O75015 |
| CD19 | P15391 |
| CD1a | P06126 |
| CD1c | P29017 |
| CD20 | P11836 |
| CD274 | Q9NZQ7 |
| CD3 | P09693 + P04234 + P07766 + P20963 |
| CD40 | P25942 |
| CD45 | P08575 |
| CD56 | P13591 |
| CD7 | P09564 |
| CD83 | Q01151 |
| CD86 | P42081 |
| CXCL10 (IP10) | P02778 |
| FS-HBP1 | O77420 |
| FS-HBP2 | O77421 |
| G-CSF | P09919 |
| GM-CSF | P04141 |
| HLA-DR | * |
| IFN-alpha2 | P01563 |
| IFNAR | P17181 + P48551 |
| IFNGR | P15260 + P38484 |
| IFN-γ | P01579 |
| IL-10 | P22301 |
| IL-12p40 | P29460 |
| IL-12p70 | P29459 + P29460 |
| IL-1-α | P01583 |
| IL-1-β | P01584 |
| IL-4 | P05112 |
| IL-6 | P05231 |
| IL-7 | P13232 |
| IL-8 | P10145 |
| Ir-LBP | B7ZDG7 |
| LIR2 | B7ZDG3 |
| LIR6 | B7ZDG7 |
| LJM111 | Q07CZ7 |
| Maxadilan | P30659 |
| Monomine | Q09JX9 |
| MS-HBP1 | O77422 |
| OMCI | Q5YD59 |
| Salp15 | Q95WZ4 |
| SHBP | Q8WSK7 |
| Sialostatin L | Q8MVB6 |
| TLR3 | O15455 |
| TLR4 | O00206 |
| TLR7 | Q9NYK1 |
| TLR8 | Q9NR97 |
| TNF-α | P01375 |
| TNFR | P20333 |
| TSGP1 | F6K8G8 |
| TSGP2 | Q8I9U1 |

* HLA database: <http://www.ebi.ac.uk/imgt/hla/>

UniProt accession numbers of proteins mentioned in the text, and not given elsewhere.
